# Relative cost-effectiveness of long-acting injectable cabotegravir versus oral pre-exposure prophylaxis in South Africa based on the HPTN 083 and HPTN 084 trials: a modelled economic evaluation and threshold analysis

**DOI:** 10.1016/S2352-3018(22)00251-X

**Published:** 2022-11-07

**Authors:** Lise Jamieson, Leigh F Johnson, Brooke E Nichols, Sinead Delany-Moretlwe, Mina C Hosseinipour, Colin Russell, Gesine Meyer-Rath

**Affiliations:** a Health Economics and Epidemiology Research Office, Department of Internal Medicine, School of Clinical Medicine, Faculty of Health Sciences, University of the Witwatersrand, Johannesburg, South Africa; b Wits Reproductive Health and HIV Institute, Faculty of Health Sciences, University of the Witwatersrand, Johannesburg, South Africa; c Department of Medical Microbiology, Amsterdam University Medical Centre, Amsterdam, Netherlands; d Centre of Infectious Disease Epidemiology and Research (CIDER), University of Cape Town, Rondebosch, Western Cape, South Africa; e Department of Global Health, Boston University School of Public Health, Boston, MA, USA; f Division of Infectious Diseases, Department of Medicine, University of North Carolina, Chapel Hill, NC, USA; g UNC Project, Lilongwe, Malawi

## Abstract

**Background:**

Long-acting injectable cabotegravir, a drug taken every 2 months, has been shown to be more effective at preventing HIV infection than daily oral tenofovir disoproxil fumarate and emtricitabine, but its cost-effectiveness in a high-prevalence setting is not known. We aimed to estimate the incremental cost-effectiveness of long-acting injectable cabotegravir compared with tenofovir disoproxil fumarate and emtricitabine in South Africa, using methods standard to government planning, and to determine the threshold price at which long-acting injectable cabotegravir is as cost-effective as tenofovir disoproxil fumarate and emtricitabine.

**Methods:**

In this modelled economic evaluation and threshold analysis, we updated a deterministic model of the South African HIV epidemic with data from the HPTN 083 and HPTN 084 trials to evaluate the effect of tenofovir disoproxil fumarate and emtricitabine and long-acting injectable cabotegravir provision to heterosexual adolescents and young women and men aged 15–24 years, female sex workers, and men who have sex with men. We estimated the average intervention cost, in 2021 US$, using ingredients-based costing, and modelled the cost-effectiveness of two coverage scenarios (medium or high, assuming higher uptake of long-acting injectable cabotegravir than tenofovir disoproxil fumarate and emtricitabine throughout) and, for long-acting injectable cabotegravir, two duration subscenarios (minimum: same pre-exposure prophylaxis duration as for tenofovir disoproxil fumarate and emtricitabine; maximum: longer duration than tenofovir disoproxil fumarate and emtricitabine) over 2022–41.

**Findings:**

Across long-acting injectable cabotegravir scenarios, 15–28% more new HIV infections were averted compared with the baseline scenario (current tenofovir disoproxil fumarate and emtricitabine roll-out). In scenarios with increased coverage with oral tenofovir disoproxil fumarate and emtricitabine, 4–8% more new HIV infections were averted compared with the baseline scenario. If long-acting injectable cabotegravir drug costs were equal to those of tenofovir disoproxil fumarate and emtricitabine for the same 2-month period, the incremental cost of long-acting injectable cabotegravir to the HIV programme was higher than that of tenofovir disoproxil fumarate and emtricitabine (5–10% *vs* 2–4%) due to higher assumed uptake of long-acting injectable cabotegravir. The cost per infection averted was $6053–6610 (tenofovir disoproxil fumarate and emtricitabine) and $4471–6785 (long-acting injectable cabotegravir). The cost per long-acting cabotegravir injection needed to be less than twice that of a 2-month supply of tenofovir disoproxil fumarate and emtricitabine to remain as cost-effective, with threshold prices ranging between $9·03 per injection (high coverage; maximum duration) and $14·47 per injection (medium coverage; minimum duration).

**Interpretation:**

Long-acting injectable cabotegravir could potentially substantially change HIV prevention. However, for its implementation to be financially feasible across low-income and middle-income countries with high HIV incidence, long-acting injectable cabotegravir must be reasonably priced.

**Funding:**

United States Agency for International Development, The Bill & Melinda Gates Foundation.

## Introduction

In 2019, South Africa had an estimated 7·8 million people living with HIV and an HIV incidence of 7·79 per 1000 population.^1^ Oral pre-exposure prophylaxis (PrEP) with the combination drug tenofovir disoproxil fumarate and emtricitabine has been shown to be effective in preventing HIV acquisition,^2^, ^3^ but there are concerns about low adherence and persistent use in many settings, including South Africa.^3^ In 2015, WHO recommended that oral PrEP be made available to people at substantial risk of acquiring HIV, followed soon after by South African guidelines.^4^ Since then, new generations of long-acting PrEP have been in development. Most recently, clinical trials (HPTN 083 and HPTN 084) conducted across the USA, Latin America, and sub-Saharan Africa have shown long-acting injectable cabotegravir to be highly efficacious in preventing HIV infection, reducing the risk of HIV acquisition by 66% (95% CI 38–82) in men who have sex with men (MSM) and transgender women, and by 89% (95% CI 68–96) in young women aged 18–45 years, compared with oral tenofovir disoproxil fumarate and emtricitabine over 12 months.^5^, ^6^ The results in young women have recently been confirmed over a 24-month period.^7^ In 2021, the US Food and Drug Administration approved long-acting injectable cabotegravir for use in populations at high risk, and in July, 2022, WHO recommended it for use in populations at substantial HIV-acquisition risk.^8^ Long-acting injectable products offer an adherence advantage over daily pill taking,^9^ and across populations at high risk, acceptability studies have shown a strong stated preference for injectable products over oral formulations.^10^, ^11^, ^12^ However, long-term effective use will require the user to maintain a visit schedule of every 2 months.

**Research in context**

**Evidence before this study**

Randomised controlled trials (HPTN 083 and HPTN 084) of long-acting injectable cabotegravir have found superior effectiveness in preventing HIV acquisition in populations at high risk compared with the standard-of-care oral pre-exposure prophylaxis (PrEP) combination drug tenofovir disoproxil fumarate and emtricitabine. These trials found a risk reduction in acquiring HIV of 66% in men who have sex with men and transgender women, and 89% in young women, compared with oral PrEP. Searching PubMed on Aug 27, 2022, for articles published in English, using the search terms (cabotegravir OR rilpivirine) AND injectable AND (prophylaxis OR PrEP OR prevention OR acceptability OR preference) AND (HIV OR human immunodeficiency virus), we found five modelling studies that evaluated the long-term effect or cost-effectiveness of the provision of long-acting injectable cabotegravir as prevention in South Africa, and found it to be cost-effective if targeted towards individuals at high risk. Two additional studies found long-acting injectable cabotegravir to be less cost-effective than oral PrEP and concluded that novel financing mechanisms might be required to make implementation cost-effective. Between the manufacturer and international organisations, the feasible minimum price of long-acting injectable cabotegravir for HIV programmes in high-burden, low-resource countries ranges from US$16 (excluding capital expenditure) to $270 per patient per year on PrEP.

**Added value of this study**

The introduction of new interventions requires careful consideration of cost as well as impact, especially in resource-limited settings. We estimated the effect and cost-effectiveness long-acting injectable cabotegravir injected every 2 months compared with daily oral tenofovir disoproxil fumarate and emtricitabine in South Africa, and found it to have reduced HIV infections and AIDS deaths by 3 times more than tenofovir disoproxil fumarate and emtricitabine. A threshold analysis estimated that the cost per long-acting injectable cabotegravir injection would need to be between $9·03 and $14·47 for it to be similarly or more cost-effective than daily oral tenofovir disoproxil fumarate and emtricitabine, and hence acceptable to the South African Government, the main funder of the South African HIV programme. This would place the cost of long-acting injectable cabotegravir at 1–2 times that of the current price for tenofovir disoproxil fumarate and emtricitabine in South Africa, with an upper limit of $101·29 per year, approximately 0·5% of the current list price in the USA.

**Implications of all the available evidence**

Our findings are timely and relevant to the decision-making process of governments in low-income and middle-income countries and donor agencies contemplating whether, and how quickly, to replace or augment oral PrEP with long-acting injectable cabotegravir. Although long-acting injectable cabotegravir has the potential to substantially change HIV prevention, for large-scale implementation in high-prevalence settings, it would first need to be affordable in these settings, and this will require a multipartner effort.

Introducing new drugs requires careful consideration of cost, cost-effectiveness, and affordability, especially in countries with severely limited resources. There have been few studies on the cost and effect of injectable PrEP in sub-Saharan Africa, and only two studies comparing injectable PrEP with oral PrEP.^13^, ^14^, ^15^, ^16^, ^17^ Previous modelling studies focusing on South Africa found that long-acting injectable cabotegravir would lead to a substantial reduction of new HIV infections compared with no PrEP.^13^, ^14^, ^15^ Of the studies evaluating cost-effectiveness, one found a risk-prioritised strategy to be cost-effective over 10 years under a threshold of 3-times gross domestic product, compared with no PrEP,^13^ and another found long-acting injectable cabotegravir to be cost-effective at a price of less than US$16 per year over 40 years under an arbitrary threshold of less than $519 per disability-adjusted life-year averted.^15^ Another two modelling studies found injectable PrEP to be less cost-effective than oral PrEP, and concluded that it might require novel financing mechanisms to be implemented.^16^, ^17^ However, these analyses were conducted before the effectiveness of long-acting injectable cabotegravir was known. Discussions of an acceptable price level for high-burden, low-resource countries range between $16 and $20 per person-year, excluding capital expenditure estimated by the Clinton Health Action Initiative, based on the costs of producing injectable contraceptives at scale in sub-Saharan Africa, and the $240–270 not-for-profit price considered by the manufacturer.

The South African Government base their decisions on affordability and impact rather than a defined cost-effectiveness threshold; in keeping with this, we aimed to establish the cost-effectiveness of novel interventions through comparison with that of already funded interventions with a similar target population, using standard methodology developed for the annual South African HIV Investment Case, which is central to HIV programme planning in South Africa.^18^ We aimed to estimate the effect and cost-effectiveness of long-acting injectable cabotegravir every 2 months compared with daily oral tenofovir disoproxil fumarate and emtricitabine in South Africa and the threshold cost that would make long-acting injectable cabotegravir similarly or more cost-effective than tenofovir disoproxil fumarate and emtricitabine.

## Methods

### Epidemiological model

In this modelled economic evaluation and threshold analysis, the effect of tenofovir disoproxil fumarate and emtricitabine and long-acting injectable cabotegravir on the HIV epidemic was estimated using Thembisa (version 4.4), a deterministic compartmental HIV transmission model of the South African HIV epidemic.^19^ The model population was stratified by age, sex, sexual experience, sexual behaviour, marital status, HIV testing history, and male circumcision status. The sexually experienced population was divided into two broad sexual risk groups: high-risk (people with a propensity for concurrent partnerships, sex work, or both); and low-risk, with the high-risk group comprising 35% of men and boys and 25% of women and girls. PrEP uptake rates varied by age, sex, and risk group. It was assumed that people taking PrEP had a 10% lower rate of condom use than individuals of the same age and sex who were not using PrEP. Individuals who acquired HIV while on PrEP were assumed to be less likely to transmit HIV than individuals who acquired HIV in the absence of regular HIV testing, as the latter individuals would remain undiagnosed and untreated for longer periods. Tenofovir disoproxil fumarate and emtricitabine effectiveness, accounting for both efficacy and adherence, was assumed to be 85% for adolescent boys and young men (aged 15–24 years) and MSM, and 65% for adolescent girls and young women (aged 15–24 years) and female sex workers.^2^, ^3^ Because long-acting injectable cabotegravir was trialled against tenofovir disoproxil fumarate and emtricitabine as a control group, we estimated the effectiveness of long-acting injectable cabotegravir compared with no PrEP by modifying the trial results, which yielded an approximate estimate of 95% effectiveness (ie, 0·95 = 1 − [1 − 0·85] × [1 − 0·66] for MSM; 0·96 = 1 – [1 – 0·65] × [1 – 0·89] for young women).^5^, ^6^ More detail on the epidemiological model, including sources for the main assumptions, are shown in the appendix (p 2).

Because this study did not include primary data from human participants, no ethical clearance was sought. No health economic analysis plan was developed.

### Scenarios and assumptions

We modelled the epidemiological impact over a 20-year period (2022–41) separately for tenofovir disoproxil fumarate and emtricitabine and long-acting injectable cabotegravir based on data from the HPTN 083 and HPTN 084 trials, including female sex workers, MSM, adolescent girls and young women (aged 15–24 years), and heterosexual adolescent boys and young men (aged 15–24 years) as target populations. We assumed two coverage levels for scaling up each PrEP technology for each population (high and medium coverage), assuming a higher uptake by users of long-acting injectable cabotegravir than users of tenofovir disoproxil fumarate and emtricitabine, based on studies showing a higher stated preference for injectable products than tenofovir disoproxil fumarate and emtricitabine.^12^, ^20^, ^21^ PrEP coverage was assumed to increase linearly over a 3 year period. On the basis of South African PrEP implementation programme data,^19^ tenofovir disoproxil fumarate and emtricitabine coverage was assumed to be low (between 0·5% and 3·2% of the relevant target populations), and the average duration on tenofovir disoproxil fumarate and emtricitabine was assumed to be 5 months for adolescent girls and young women and adolescent boys and young men, and 11 months for MSM. There was no tenofovir disoproxil fumarate and emtricitabine uptake in long-acting injectable cabotegravir scenarios. We assumed that a 1-month supply of tenofovir disoproxil fumarate and emtricitabine at last visit provided an additional month of protection. For long-acting injectable cabotegravir, the average duration in the programme was modelled under two subscenarios: (1) a minimum duration scenario, in which users remain in the programme for a similar time as they would on tenofovir disoproxil fumarate and emtricitabine; and (2) a maximum duration scenario, in which users remain on PrEP for longer—ie, 12 months (for adolescent boys and young men and adolescent girls and young women) or 24 months (for MSM). Although annual PrEP initiation rates for the tenofovir disoproxil fumarate and emtricitabine and the long-acting injectable cabotegravir minimum duration scenario were based on the assumed coverage for each population, the initiation rates for the long-acting injectable cabotegravir maximum duration scenario were fixed to the same rates as the long-acting injectable cabotegravir minimum duration scenario, but with longer duration in the PrEP programme (resulting in higher coverage than in the minimum duration scenario). In accordance with the trial data, we assumed seven injections per year on long-acting injectable cabotegravir, at initiation and at months 1, 3, 5, 7, 9, and 11 (table 1).

**Table 1:** Key modelling assumptions on coverage, duration, and effectiveness of PrEP scenarios

		**Baseline (tenofovir disoproxil fumarate and emtricitabine only)**	**Tenofovir disoproxil fumarate and emtricitabine**	**Long-acting injectable cabotegravir minimum duration**	**Long-acting injectable cabotegravir maximum duration**	**Data source**
Coverage scenarios, % coverage in population						
	Baseline	3% (FSW); 1% (MSM); 0·5% (AGYW); 0% (ABYM)	··	··	··	Tenofovir disoproxil fumarate and emtricitabine: PrEP implementation data from South African National Department of Health
	High coverage	··	30% (FSW, MSM); 10% (AGYW, ABYM)	50% (FSW, MSM); 40% (AGYW); 20% (ABYM)	67% (FSW, MSM); 60% (AGYW); 35% (ABYM)	Tenofovir disoproxil fumarate and emtricitabine: PrEP implementation data from South African National Department of Health. Long-acting injectable cabotegravir: informed by acceptability and preference studies^19^, ^22^, ^23^
	Medium coverage	··	15% (FSW, MSM); 5% (AGYW, ABYM)	25% (FSW, MSM); 20% (AGYW); 10% (ABYM)	40% (FSW, MSM); 35% (AGYW); 20% (ABYM)	Tenofovir disoproxil fumarate and emtricitabine: PrEP implementation data from South African National Department of Health. Long-acting injectable cabotegravir: informed by acceptability and preference studies^19^, ^22^, ^23^
Duration in PrEP programme, months		5 (FSW, AGYW, ABYM); 11 (MSM)	5 (FSW, AGYW, ABYM); 11 (MSM)	5 (FSW, AGYW, ABYM); 11 (MSM)	12 (FSW, AGYW, ABYM); 24 (MSM)	Tenofovir disoproxil fumarate and emtricitabine: Chiu and colleagues.^24^ Long-acting injectable cabotegravir: assumed values
Additional protection since last visit in PrEP programme		1 month (all populations)	1 month (all populations)	3 months (all populations)	3 months (all populations)	Tenofovir disoproxil fumarate and emtricitabine: assumed values. Long-acting injectable cabotegravir: Marzinke and colleagues^25^
Total protection duration, months		6 (FSW, AGYW, ABYM); 12 (MSM)	6 (FSW, AGYW, ABYM); 12 (MSM)	8 (FSW, AGYW, ABYM); 14 (MSM)	15 (FSW, AGYW); 15 (ABYM); 27 (MSM)	Values estimated from duration in PrEP programme plus additional protection
Effectiveness		65% (FSW, AGYW); 85% (ABYM, MSM)	65% (FSW, AGYW); 85% (ABYM, MSM)	95% (all populations)	95% (all populations)	Tenofovir disoproxil fumarate and emtricitabine: Fonner and colleagues,^3^ Cherish and colleagues,^26^ and Ross and colleagues.^27^ Long-acting injectable cabotegravir: Cheng and colleagues^12^ and Glaubius and colleagues^13^

ABYM=adolescent boys and young men. AGYW=adolescent girls and young women. FSW=female sex workers. MSM=men who have sex with men. PrEP=pre-exposure prophylaxis.

### Cost and cost-effectiveness

Costs were analysed from the perspective of the provider, the South African Government, and reported in 2021 US$, using the average exchange rate of January to October, 2021 (14·61 South African rand = $1).^28^ The average cost of PrEP provision across scenarios was estimated using an ingredients-based approach; the full methodology has been described elsewhere.^29^ Briefly, PrEP is provided in primary health-care clinics and includes rapid HIV testing, counselling, provision of condoms, syndromic screening for sexually transmitted infections with treatment referral, adherence counselling, as well as training, outreach, mobilisation, monitoring, and evaluation costs. We varied costs between the first versus follow-up years (where applicable) and populations, capturing differences in HIV and sexually transmitted infection prevalence and need for pregnancy tests.

Because long-acting injectable cabotegravir is not currently available in South Africa, the cost of its provision was based on that of tenofovir disproxil fumarate and emtricitabine and adjusted by increasing professional nurse time for injection administration and removing creatinine testing (required for tenofovir-disoproxil-fumarate-based PrEP only). We allowed for an oral cabotegravir lead-in, assuming 20% of individuals who initiated into the programme would opt to start with oral cabotegravir for the first month. The cost structure of long-acting injectable cabotegravir provision is shown in the appendix (p 7). Because the cost of injectable cabotegravir in the public health-care sector is currently unknown, we varied the price of long-acting injectable cabotegravir between 1 and 5 times the 2-monthly price of oral tenofovir disoproxil fumarate and emtricitabine. Finally, we solved for the optimal price at which long-acting injectable cabotegravir is as cost-effective as tenofovir disoproxil fumarate and emtricitabine (table 2).

**Table 2:** Average cost of tenofovir disoproxil fumarate and emtricitabine and long-acting injectable cabotegravir provision per person initiated* across scenarios and target populations

	**Female sex workers**	**Adolescent girls and young women**	**Heterosexual men**	**Men who have sex with men (first year)**	**Men who have sex with men (follow-up year)**
**Tenofovir disoproxil fumarate and emtricitabine**					
Total cost	$78	$77	$76	$116	NA
Drugs	$28 (36%)	$28 (36%)	$28 (37%)	$56 (48%)	NA
Laboratories	$16 (21%)	$16 (21%)	$15 (20%)	$15 (13%)	NA
Consumables	$3 (4%)	$2 (3%)	$2 (3%)	$4 (3%)	NA
Staff	$23 (29%)	$22 (29%)	$22 (29%)	$29 (25%)	NA
Overheads	$9 (12%)	$8 (10%)	$8 (11%)	$11 (9%)	NA
**Long-acting injectable cabotegravir minimum duration**					
Total cost	$81	$80	$78	$122	NA
Drugs	$37 (46%)	$37 (46%)	$37 (47%)	$65 (53%)	NA
Laboratories	$10 (12%)	$9 (11%)	$8 (10%)	$9 (8%)	NA
Consumables	$1 (1%)	$1 (1%)	$1 (1%)	$1 (1%)	NA
Staff	$29 (36%)	$28 (35%)	$28 (36%)	$39 (32%)	NA
Overheads	$5 (6%)	$5 (6%)	$5 (6%)	$7 (6%)	NA
**Long-acting injectable cabotegravir maximum duration**					
Total cost	$137	$137	$134	$131	$105
Drugs	$67 (49%)	$67 (49%)	$67 (50%)	$67 (51%)	$56 (53%)
Laboratories	$15 (11%)	$16 (12%)	$14 (10%)	$11 (8%)	$6 (6%)
Consumables	$2 (1%)	$1 (1%)	$1 (1%)	$1 (1%)	$1 (1%)
Staff	$46 (34%)	$44 (32%)	$44 (33%)	$44 (34%)	$35 (33%)
Overheads	$8 (6%)	$8 (6%)	$8 (6%)	$8 (6%)	$6 (6%)

Totals might not add up to the sum of the subcomponents due to rounding of all figures to the nearest $1. All values are in 2021 US dollars ($). NA=not applicable.

* Duration in the pre-exposure prophylaxis programme differed by population, intervention (tenofovir disoproxil fumarate and emtricitabine or long-acting injectable cabotegravir), and scenario: tenofovir disoproxil fumarate and emtricitabine and long-acting injectable cabotegravir minimum duration scenarios (5 months for adolescent girls, young women, female sex workers, and heterosexual men; 11 months for men who have sex with men); long-acting injectable cabotegravir maximum duration scenarios (12 months for adolescent girls, young women, female sex workers, and heterosexual men; 24 months for men who have sex with men).

We analysed cost-effectiveness over a 20 year period (2022–41), compared with a baseline of currently available HIV interventions in South Africa, including the current tenofovir disoproxil fumarate and emtricitabine programme, and high coverages for condom provision, HIV testing services, and medical male circumcision. This allowed us to ascertain the effect of a reduction in HIV incidence on the need for subsequent antiretroviral therapy (ART), in addition to existing prevention interventions. The estimation of HIV programme costs followed the same approach as the South African HIV Investment Case.^24^ We report on cost-effectiveness as cost per HIV infection averted and per life-year saved, the metrics most relevant to the decision space of the South African Government, in line with the principles of the International Decision Support Initiative reference case for economic evaluation.^30^ To facilitate the use of the results in informing government budgets and an acceptable threshold price for long-acting injectable cabotegravir for use in the South African Government's negotiations with manufacturers, costs and effects are presented undiscounted over the period modelled.

### Sensitivity analysis

We reproduced the main analysis with four modifications: (1) an injection schedule of every 3 months instead of every 2 months for long-acting injectable cabotegravir, based on pharmacokinetic data suggesting that longer protection is feasible;^31^ (2) assuming long-acting injectable cabotegravir coverage would be the same as that of tenofovir disoproxil fumarate and emtricitabine scenarios; (3) testing different discount rates instead of 0% (3·00%, 4·75%, and 6·00%), and (4) assuming PCR testing in the HIV diagnostic algorithm. Furthermore, we evaluated the effect of the uncertainty around the following additional key model parameters on the results: intervention effectiveness for both tenofovir disoproxil fumarate and emtricitabine and long-acting injectable cabotegravir; reduction in condom use while on PrEP; annual initiation rate for tenofovir disoproxil fumarate and emtricitabine; relative annual initiation rate for long-acting injectable cabotegravir (to ensure a value consistently higher than the corresponding tenofovir disoproxil fumarate and emtricitabine scenario); relative rate of PrEP initiation in heterosexual people who are at low risk; and non-drug costs. To do this, we conducted a probabilistic sensitivity analysis using Monte Carlo simulation with 1000 model runs, sampling values from predetermined distributions for each model run (appendix p 11). We fixed the cost of the long-acting injectable cabotegravir drug at twice the cost of the tenofovir disoproxil fumarate and emtricitabine drug for this analysis and report median estimates, 2·5th and 97·5th percentiles, and partial rank correlation coefficients quantifying the sensitivity of central model results to changes in these parameters.

### Role of the funding source

The funders of the study had no role in study design, data collection, data analysis, data interpretation, or writing of the report.

## Results

**Figure 1:**
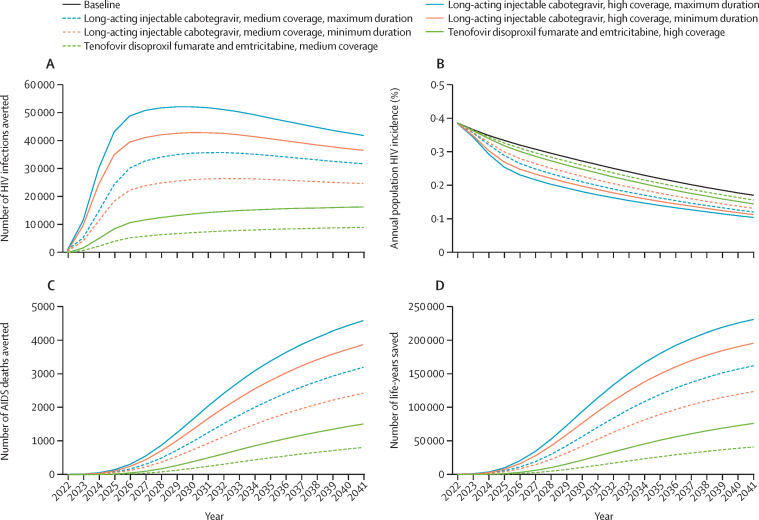
Effect of long-acting injectable cabotegravir and oral pre-exposure prophylaxis (tenofovir disoproxil fumarate and emtricitabine) on HIV infections and deaths, 2022–41 (A) Annual HIV infections averted. (B) Annual population HIV incidence. (C) Annual AIDS deaths averted. (D) Annual life-years saved over baseline (total population size over time horizon was approximately 60–73 million). Long-acting injectable cabotegravir and tenofovir disoproxil fumarate and emtricitabine are modelled under two coverage scenarios (high and medium); long-acting injectable cabotegravir is additionally modelled under both a minimum and maximum scenario, as described in table 1.

Across scenarios, long-acting injectable cabotegravir had a large effect on new HIV infections in South Africa, with a maximum of 52 000 infections averted per year across all scenarios compared with baseline in the high coverage, maximum duration scenario, 42 800 infections averted per year in the high coverage, minimum duration scenario, 35 600 infections averted per year in the medium coverage, maximum duration scenario, and 26 400 infections averted per year in the medium coverage, minimum duration scenario—a reduction of 15–28% of infections over 20 years (figure 1A). Tenofovir disoproxil fumarate and emtricitabine averted 16 300 infections annually in the high coverage scenario and 9000 infections annually in the medium coverage scenario, an overall reduction of 4–8% of infections over 20 years. From 2030, the number of HIV infections averted decreased in all long-acting injectable cabotegravir scenarios, indicating intervention saturation in the context of declining HIV incidence. HIV incidence was projected to decrease to 0·17% in 2041 under the baseline scenario (figure 1B). Long-acting injectable cabotegravir reduced incidence to between 0·10% (high coverage, maximum duration) and 0·13% (medium coverage, minimum duration) by 2041; whereas, under the tenofovir disoproxil fumarate and emtricitabine scenarios, HIV incidence declined to 0·14% (high coverage) and 0·16% (medium coverage) by 2041.

**Figure 2:**
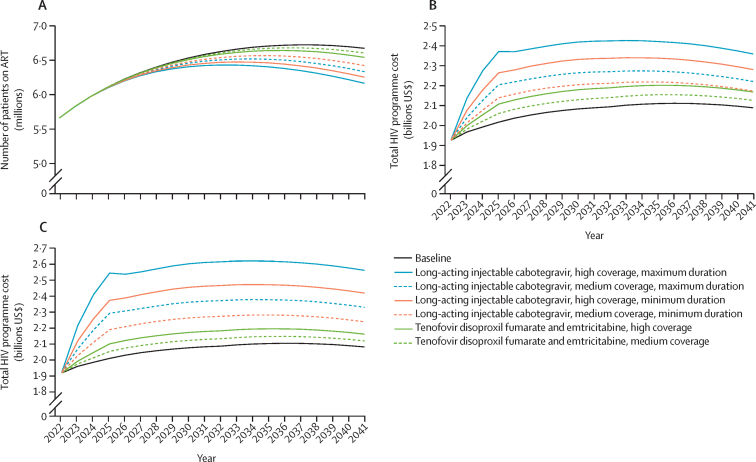
Effect of long-acting injectable cabotegravir and oral pre-exposure prophylaxis (tenofovir disoproxil fumarate and emtricitabine) on patients on ART and HIV programme cost, 2022–41 (A) Annual total patients on ART. (B) Total HIV programme cost if long-acting injectable cabotegravir drug price was the same as tenofovir disoproxil fumarate and emtricitabine. (C) Total HIV programme cost if long-acting injectable cabotegravir drug price was twice that of tenofovir disoproxil fumarate and emtricitabine. ART=antiretroviral therapy.

Long-acting injectable cabotegravir was projected to avert between 21 500 (2·0%) and 43 400 (4·0%) AIDS deaths; whereas, tenofovir disoproxil fumarate and emtricitabine was projected to reduce AIDS deaths by 12 400 (1·2%) in the high coverage scenario and 6500 (0·6%) in the medium coverage scenario over the same time horizon (figure 1C). Additionally, long-acting injectable cabotegravir saved between 57 600 life-years (medium coverage, minimum duration) and 115 700 life-years (high coverage, maximum duration) on average per year; whereas, tenofovir disoproxil fumarate and emtricitabine saved between 32 700 life-years (high coverage) and 17 000 life-years (medium coverage) per year (figure 1D). There was a 4–8% reduction in the number of people on ART by 2041 in the long-acting injectable cabotegravir scenarios over baseline, compared with a 1–2% reduction for the tenofovir disoproxil fumarate and emtricitabine scenarios—a relative 3-to-5-fold reduction in the number of people on ART in the long-acting injectable cabotegravir scenarios compared with tenofovir disoproxil fumarate and emtricitabine (figure 2A). Although the reduction in ART need will result in a reduction in HIV programme cost, the incremental cost of providing tenofovir disoproxil fumarate and emtricitabine or long-acting injectable cabotegravir at the assumed coverage levels will be more than the savings from the reduction in ART within the next 20 years, increasing total programme cost by between 5% (medium coverage) and 10% (high coverage) under long-acting injectable cabotegravir, or between 2% (medium coverage) and 4% (high coverage) under tenofovir disoproxil fumarate and emtricitabine (figure 2B; appendix p 12). The proportion of the total cost of the HIV programme spent on HIV prevention would be 5% under the baseline scenario, increasing to 8–10% under tenofovir disoproxil fumarate and emtricitabine scale-up, and to 11–14% (medium coverage), and 17–20% (high coverage) under long-acting injectable cabotegravir. This proportion would further increase at higher prices of long-acting injectable cabotegravir (appendix p 12) but would still be lower than the UNAIDS recommended 25% to be spent on prevention.^22^

Under increased tenofovir disoproxil fumarate and emtricitabine coverage, the incremental cost-effectiveness ratio was $2309 per life-year saved (medium coverage) and $2498 per life-year saved (high coverage; table 3). For long-acting injectable cabotegravir to remain as cost-effective as tenofovir disoproxil fumarate and emtricitabine, the cost of the drug would need to be between 1-fold and 2-fold that of tenofovir disoproxil fumarate and emtricitabine (2 months’ supply). We estimated the threshold price for long-acting injectable cabotegravir per injection to be between $9·03 (high coverage, maximum duration) and $14·47 (medium coverage, minimum duration) if it was to remain as cost-effective as tenofovir disoproxil fumarate and emtricitabine (appendix p 12).

**Table 3:** Effect and cost-effectiveness of long-acting injectable cabotegravir compared with baseline* and oral tenofovir disoproxil fumarate and emtricitabine compared with baseline, over a 20 year period (2022–41)

			**Total cost of the HIV programme**		**Incremental cost effectiveness**		**New HIV infections**		**Life-years lost due to AIDS**	
			Cost (billions US$)	Incremental cost over baseline	Cost per infection averted (US$)	Cost per life-year saved (US$)	Number (millions)	Proportion averted over baseline	Number (millions)	Proportion saved over baseline
Baseline			$41·29	··	··	··	3·02	··	37·34	··
Medium PrEP coverage										
	Tenofovir disoproxil fumarate and emtricitabine		$42·08	2%	$6053	$2309	2·89	4%	37·00	1%
	Long-acting injectable cabotegravir minimum duration									
		Cost same as tenofovir disoproxil fumarate and emtricitabine	$43·25	5%	$4471	$1705	2·58	15%	36·19	3%
		Cost 2 × greater than tenofovir disoproxil fumarate and emtricitabine	$44·46	8%	$7211	$2751	2·58	15%	36·19	3%
		Cost 3 × greater than tenofovir disoproxil fumarate and emtricitabine	$45·66	11%	$9952	$3796	2·58	15%	36·19	3%
		Cost 4 × greater than tenofovir disoproxil fumarate and emtricitabine	$46·86	13%	$12 692	$4842	2·58	15%	36·19	3%
		Cost 5 × greater than tenofovir disoproxil fumarate and emtricitabine	$48·07	16%	$15 433	$5887	2·58	15%	36·19	3%
	Long-acting injectable cabotegravir maximum duration									
		Cost same as tenofovir disoproxil fumarate and emtricitabine	$44·31	7%	$5157	$1978	2·44	19%	35·81	4%
		Cost 2 × greater than tenofovir disoproxil fumarate and emtricitabine	$46·24	12%	$8447	$3240	2·44	19%	35·81	4%
		Cost 3 × greater than tenofovir disoproxil fumarate and emtricitabine	$48·16	17%	$11 737	$4501	2·44	19%	35·81	4%
		Cost 4 × greater than tenofovir disoproxil fumarate and emtricitabine	$50·09	21%	$15 027	$5763	2·44	19%	35·81	4%
		Cost 5 × greater than tenofovir disoproxil fumarate and emtricitabine	$52·02	26%	$18 317	$7025	2·44	19%	35·81	4%
High PrEP coverage										
	Tenofovir disoproxil fumarate and emtricitabine		$42·92	4%	$6610	$2498	2·78	8%	36·68	2%
	Long-acting injectable cabotegravir minimum duration									
		Cost same as tenofovir disoproxil fumarate and emtricitabine	$45·42	10%	$5779	$2145	2·31	24%	35·41	5%
		Cost 2 × greater than tenofovir disoproxil fumarate and emtricitabine	$47·83	16%	$9147	$3394	2·31	24%	35·41	5%
		Cost 3 × greater than tenofovir disoproxil fumarate and emtricitabine	$50·24	22%	$12 515	$4644	2·31	24%	35·41	5%
		Cost 4 × greater than tenofovir disoproxil fumarate and emtricitabine	$52·64	27%	$15 882	$5894	2·31	24%	35·41	5%
		Cost 5 × greater than tenofovir disoproxil fumarate and emtricitabine	$55·05	33%	$19 250	$7144	2·31	24%	35·41	5%
	Long-acting injectable cabotegravir maximum duration									
		Cost same as tenofovir disoproxil fumarate and emtricitabine	$47·10	14%	$6785	$2510	2·17	28%	35·03	6%
		Cost 2 × greater than tenofovir disoproxil fumarate and emtricitabine	$50·63	23%	$10 915	$4038	2·17	28%	35·03	6%
		Cost 3 × greater than tenofovir disoproxil fumarate and emtricitabine	$54·16	31%	$15 045	$5566	2·17	28%	35·03	6%
		Cost 4 × greater than tenofovir disoproxil fumarate and emtricitabine	$57·70	40%	$19 175	$7094	2·17	28%	35·03	6%
		Cost 5 × greater than tenofovir disoproxil fumarate and emtricitabine	$61·23	48%	$23 305	$8622	2·17	28%	35·03	6%

Long-acting injectable cabotegravir drug cost relative to tenofovir disoproxil fumarate and emtricitabine drug cost is for the drug only, excluding cost of provision (staff, laboratory monitoring, consumables, and overheads). Costs are in 2021 US$. Rounding might have caused some discrepancies in the calculations of some values. NA=not applicable. PrEP=pre-exposure prophylaxis.

* Baseline scenario: current roll-out of tenofovir disoproxil fumarate and emtricitabine as standard of care PrEP (see table 1 for comparative coverage levels by population).

The cost threshold and hence acceptable price level for long-acting injectable cabotegravir would remain similar if it was administered every 3 months rather than every 2 months (appendix p 13), or had the same coverage as tenofovir disoproxil fumarate and emtricitabine (although a higher price could be accepted under the minimum duration scenario due to increased effectiveness alone; appendix p 14), and in discounted analyses (appendix pp 15–17). Including PCR testing in the HIV testing algorithm would increase its implementation cost by approximately 60% (annual PCR testing) or approximately 160% (PCR testing every 2 months) and reduce the threshold price to $0·49–6·14 per injection (5–65% of tenofovir disoproxil fumarate and emtricitabine cost; appendix p 18). When accounting for uncertainty, the median HIV infections averted over the 20 year period, compared with baseline, was 238 000 (IQR 15 600–400 000) for tenofovir disoproxil fumarate and emtricitabine, 472 300 (46 700–734 800) for long-acting injectable cabotegravir with a minimum duration, and 614 000 (81 800–873 700) for long-acting injectable cabotegravir with a maximum duration (appendix p 20). Even under the assumption that the cost of the long-acting injectable cabotegravir injectable was twice that of tenofovir disoproxil fumarate and emtricitabine, 25% of simulations under the long-acting injectable cabotegravir minimum duration scenario and 14% of simulations under the long-acting injectable cabotegravir maximum duration scenario were more cost-effective than the corresponding tenofovir disoproxil fumarate and emtricitabine simulation (appendix p 23), with the 95% CI of the cost threshold price for long-acting injectable cabotegravir per injection ranging between $8·80 and $30·80 (appendix p 23). Cost per HIV infection averted was most sensitive to PrEP efficacy, relative rate of PrEP uptake by those at low risk of HIV, PrEP initiation rates, and the non-drug costs of PrEP provision; whereas, HIV infections averted were most sensitive to PrEP initiation rates and PrEP efficacy (appendix p 22).

## Discussion

Our analysis used a well validated model of the South African HIV epidemic to show that long-acting injectable cabotegravir could avert 3 times more new HIV infections and save 3 times more life-years over 20 years compared with tenofovir disoproxil fumarate and emtricitabine under a range of different scenarios. This estimated impact of long-acting injectable cabotegravir on HIV infections is similar to other modelling studies.^13^, ^14^, ^15^ With a higher assumed uptake, the incremental cost of long-acting injectable cabotegravir to the HIV programme is likely to be more than that of further scaling up tenofovir disoproxil fumarate and emtricitabine. Our threshold analysis determined that for our given coverage and duration assumptions, the cost of long-acting injectable cabotegravir would need to be between $9·03 per injection ($63·21 per year, assuming seven injections per year in accordance with the trial—ie, at initiation and months 1, 3, 5, 7, 9, and 11) and $14·47 ($101·29 per year) to be as cost-effective as tenofovir disoproxil fumarate and emtricitabine, with a wider range once uncertainty was factored in ($8·80–30·80 per injection). A strength of our analysis is the certainty around injectable PrEP effectiveness, as previous modelling, conducted before the release of trial results,^5^, ^6^ still incorporated substantial uncertainty. Our analysis also comprehensively modelled the full cost of the South African HIV response, comprehensively assessing the cost savings associated with more effective PrEP.

Of note is the low cost of the fixed-dose combination drug, tenofovir disoproxil fumarate and emtricitabine, in South Africa, at $4·70 per month, or $56·39 per year for the drug alone, mostly due to the fact that tenofovir disoproxil fumarate and emtricitabine is a generic formulation and part of first-line ART in the world's largest ART programme, allowing for a substantial cost reduction. By contrast, long-acting injectable cabotegravir, as a recently developed drug, is protected under patent laws until 2031, and is currently sold at $22 200 per year in the USA, more than 200 times the threshold price identified in this analysis.^23^ Even given the uncertainty in our analysis, it is clear that unless dramatic price reductions occur, long-acting injectable cabotegravir will not be an option for HIV prevention for those at highest risk of HIV in low-income and middle-income countries. Options for these price reductions include the recently agreed voluntary licences through the Medicines Patent Pool, a buy-down similar to that establishing a market for HIV self-testing, or a combination of these factors. However, given that both factors depend on the availability of a fairly large target market, additional financial support for implementation and demand creation might be required in order to scale the intervention up quickly enough for effects as large as we have projected to occur.

There are several limitations to this analysis. First, although we have programme data on the uptake of tenofovir disoproxil fumarate and emtricitabine, the real-world uptake of long-acting injectable cabotegravir is unknown until the drug is implemented widely. We assumed uptake of long-acting injectable cabotegravir would be higher than that of tenofovir disoproxil fumarate and emtricitabine in our main analysis and sensitivity analyses, on the basis of acceptability and preference studies;^12^, ^20^, ^21^ however, these assumptions might not necessarily translate into real-world choices. Nevertheless, long-acting injectable cabotegravir has been shown to be significantly more effective in preventing HIV infection than tenofovir disoproxil fumarate and emtricitabine,^7^ and if long-acting injectable cabotegravir uptake remains at current levels of tenofovir disoproxil fumarate and emtricitabine uptake, we can still expect large effects on averting HIV infections. Similarly, duration of effective use of long-acting injectable cabotegravir is unknown; however, we consider it a reasonable assumption that duration on long-acting injectable cabotegravir would, at a minimum, be the same as that of tenofovir disoproxil fumarate and emtricitabine. Second, in the absence of bottom-up cost data, we used an ingredients-based approach to cost PrEP provision. Although this method could have underestimated or overestimated the incremental cost required, the cost-of-service provision in a national roll-out would probably be similar between these interventions, with drug cost being the main difference. We also assumed rapid HIV testing across both tenofovir disoproxil fumarate and emtricitabine and long-acting injectable cabotegravir programmes; a change in the diagnostic approach to include PCR testing in the long-acting injectable cabotegravir programme, to sufficiently detect the presence of virus suppressed by long-acting injectable cabotegravir,^25^ would increase its implementation cost substantially, reducing the long-acting injectable cabotegravir price to less than $6·14 per injection. Third, we modelled PrEP provision to different populations and assumed the relative uptake between these groups to remain constant across scenarios. Although selectively targeting particular subpopulations might be more cost-effective, we do expect the same higher uptake in adolescent girls and young women, female sex workers, and MSM as in the current tenofovir disoproxil fumarate and emtricitabine programme. In particular, young women already using widely accepted injectable contraceptives might find long-acting injectable cabotegravir more attractive than other groups.^26^ Fourth, in our current model framework we are not able to model the effect of both tenofovir disoproxil fumarate and emtricitabine and long-acting injectable cabotegravir simultaneously, when in reality there might be a mix between these two (and future) PrEP technologies. Typically, with each added modality, total demand increases, as seen with other products, such as contraceptives for women.^27^ However, the purpose of our analysis was to determine the price threshold for long-acting injectable cabotegravir relative to tenofovir disoproxil fumarate and emtricitabine, for which an analysis including both modalities was not required. Fifth, adherence to injectable PrEP might be different from that in a clinical trial setting, and this might compromise effectiveness. Further data on the effectiveness of injectable PrEP in the pharmacokinetic tail-phase after cessation of injections will be required in order to realistically model the consequences of such adherence challenges. Finally, a concern could be the potential for an increased risk of drug resistance from acquired infection after stopping the injectable compared with oral PrEP, because of the longer tail-phase. Given widespread use of dolutegravir-based ART regimens, this could undermine the effectiveness of ART.^18^ Allowing for such reductions in ART efficacy in our model would offset the cost savings associated with injectable PrEP as more individuals would require more expensive second-line treatment, thus lowering the cost threshold required to achieve similar cost-effectiveness to oral PrEP. Long-acting injectable cabotegravir studies have not observed drug resistance arising specifically during the tail-phase;^5^, ^6^, ^25^, ^32^ however, this could be a potential, but probably rare, concern in future with higher uptake of long-acting injectable cabotegravir.

Although we show long-acting injectable cabotegravir to be superior in its effect on the HIV epidemic in South Africa, the budget impact could be substantially more than for tenofovir disoproxil fumarate and emtricitabine if there is a higher uptake, especially if the drug cost per year is more than that of tenofovir disoproxil fumarate and emtricitabine. Preference studies have suggested that long-acting injectable cabotegravir could be a preferred prevention option over tenofovir disoproxil fumarate and emtricitabine, including in South Africa; real-world uptake and preference can only be assessed once it is available for use.^12^, ^33^ However, although long-acting injectable cabotegravir has the potential to change HIV prevention, for large-scale implementation across low-income and middle-income countries, it first needs to be affordable, and lessons learned from oral PrEP programmes show that scale-up and demand creation should be coordinated between all partners and should be fast enough to build momentum and yield results as high as those projected here.

## Data sharing

A fully parameterised Microsoft Excel version of Thembisa is available on www.thembisa.org.

## Declaration of interests

We declare no competing interests.
