# Supplementary material for: Relative cost-effectiveness of long-acting injectable cabotegravir versus oral pre-exposure prophylaxis in South Africa based on the HPTN 083 and HPTN 084 trials: a modelled economic evaluation and threshold analysis
Source: Lancet HIV. 2022 Nov 7;9(12):e857–67. doi: 10.1016/S2352-3018(22)00251-X (PMC9708606; doi:10.1016/S2352-3018(22)00251-X)
Supplement: Supplementary appendix [file mmc1.pdf]

THE LANCET
HIV

## Supplementary appendix

This appendix formed part of the original submission and has been peer reviewed. We post it as supplied by the authors.

Supplement to: Jamieson L, Johnson LF, Nichols BE, et al. Relative cost-effectiveness of long-acting injectable cabotegravir versus oral pre-exposure prophylaxis in South Africa based on the HPTN 083 and HPTN 084 trials: a modelled economic evaluation and threshold analysis. *Lancet HIV* 2022; published online Nov 7. https://doi.org/10.1016/S2352-3018(22)00251-X.

Appendix to:

# Relative cost-effectiveness of long-acting injectable cabotegravir versus oral pre-exposure prophylaxis in South Africa based on the HPTN 083 and 084 trials: a modelled economic evaluation and threshold analysis

Lise Jamieson*, MSc, Leigh F. Johnson, PhD, Brooke E. Nichols, PhD, Prof Sinead Delany-Moretlwe, PhD, Prof Mina C. Hosseinipour, MD, Prof Colin Russell, PhD, Gesine Meyer-Rath, PhD

Health Economics and Epidemiology Research Office, Department of Internal Medicine, School of Clinical Medicine, Faculty of Health Sciences, University of the Witwatersrand, Johannesburg, South Africa (LJ, BEN, GMR)

Department of Medical Microbiology, Amsterdam University Medical Centre, Amsterdam, The Netherlands (LJ, BEN, CR)

Centre of Infectious Disease Epidemiology and Research (CIDER), University of Cape Town, Rondebosch, Western Cape, South Africa (LFJ)

Department of Global Health, Boston University School of Public Health, Boston, Massachusetts, USA (BEN, CR, GMR)

Wits RHI, Faculty of Health Sciences, University of the Witwatersrand, Johannesburg, South Africa (SDM)

University of North Carolina, Chapel Hill, NC, USA (MCH)

UNC Project, Lilongwe, Malawi (MCH)

**Additional details regarding the epidemiological model**

A more detailed description of the Thembisa model (version 4·4) is provided elsewhere·[1]· Here we provide a brief overview of the assumptions most relevant to the current paper·

**Modelling of sexual risk behaviour**

Thembisa is an integrated demographic and HIV model of the South African population. The demographic component of the model stratifies the population by sex and single year of age. There are two broadly defined risk groups: the 'high-risk' group comprises individuals who have a propensity for concurrent partners and/or commercial sex, and the 'low-risk' group consists of individuals who are serially monogamous and never engage in commercial sex. Within these two broad risk groups there are several sub-groups, defined in terms of sexual experience (virgin/sexually experienced), marital status, and spouse risk group (in the case of married individuals). Female sex workers (FSWs) are modelled as a sub-group within the unmarried high-risk female group, with rates of entry into sex worker being calculated to be sufficient to meet the assumed male demand for commercial sex, and rates of exit from sex work being calculated on the assumption of a three-year average duration of sex work.[2–4] Men who have sex with men (MSM) are modelled as a sub-group of unmarried sexually-experienced men; due to high rates of heterosexual activity reported by MSM, it is assumed that 30% of sexual contacts are with female partners.[5–7] Due to low rates of marriage among South African MSM[5,8,9] and prevailing stigma around same-sex relationships, it is assumed for simplicity that MSM only marry female partners..

Rates of sexual debut depend on age, sex and risk group. After beginning sexual activity, three types of relationship are modelled: once-off contacts between sex workers and clients, short-term (non-cohabiting) relationships, and long-term (marital or cohabiting) relationships. Rates of marriage and union dissolution are assumed to vary by age and sex, based on calibration of the model to marriage prevalence data from censuses and community surveys.[10] Rates of male contact with sex workers are assumed to depend on age and marital status, with rates being highest among unmarried men and men in their thirties. Rates of short-term partnership formation depend on age, sex, risk group and marital status (low-risk individuals, by definition, do not engage in short-term relationships while married), and rates of male short-term partnership formation are calculated to be consistent with female rates, given assumptions about the mean and standard deviation of age differences in short-term relationships. Assumptions about the assortativeness of mixing determine the proportion of high-risk individuals who select partners in the low-risk group and vice versa, for both short-term and long-term relationships.

Coital frequencies are specified on a monthly basis for long-term relationships, and on a per-partnership basis for short-term relationships. Rates of condom use are assumed to depend on age, sex and relationship type, and are assumed to have increased substantially over the 1995-2010 period in response to condom promotion programmes.[11] In addition, condom use is assumed to increase after HIV diagnosis and after ART initiation.

**Modelling of HIV transmission**

The HIV epidemic is seeded in 1985 with an initial HIV prevalence in high-risk individuals aged 15-49. Thereafter the epidemic spreads based on assumptions about the probability of HIV transmission per unprotected sex act. This transmission probability varies in relation to the type of relationship (highest for short-term and MSM relationships), the sex of the susceptible partner, the circumcision status of the susceptible male partner, the HIV stage of the HIV-positive partner (highest during the acute stage of HIV infection and when untreated with a CD4 count of <200 cells/μl), and whether the HIV-positive partner is treated (transmission from treated individuals further depends on assumptions about prevailing levels of viral suppression). Condoms are assumed to be 95% effective in preventing transmission[12,13] and men who are circumcised are assumed to be 60% less likely to acquire HIV during heterosexual sex than uncircumcised men.[14] The numbers of HIV-positive individuals and the proportions in different HIV stages are updated at monthly time steps.

**Modelling of HIV disease progression**

In the absence of treatment, adults who acquire HIV are assumed to progress through five stages of HIV disease: an initial acute stage (lasting an average of 3 months) and four subsequent stages that are defined in terms of CD4 count (≥500, 350-499, 200-349 and <200 cells/μl). Mortality due to AIDS is assumed to occur at CD4 counts of <350 cells/μl. Three types of HIV testing are modelled: testing of pregnant women attending antenatal clinics, testing of patients with opportunistic infection symptoms, and other 'general' HIV testing, with testing rates being assumed to change over time on the basis of routine testing and survey data.[15,16] Following HIV diagnosis, a proportion of the newly diagnosed are assumed to initiate ART immediately (if eligible) and the balance are

assumed to defer ART initiation. Rates of ART initiation after diagnosis change over time based on changing ART eligibility criteria, and are assumed to be lower at higher CD4 counts. Individuals who start ART are classified according to their baseline CD4 count and the time since first ART initiation; at each ART duration it is further assumed that a certain proportion of patients who have initiated ART are currently interrupting ART (these proportions are calculated from assumptions about annual ART interruption rates and average durations of interruption). Mortality rates in treated patients are assumed to depend on both the baseline CD4 count and the duration since first ART initiation.

**Model calibration**

The model is calibrated to a number of data sources:

- HIV prevalence data from national antenatal surveys (1991-2015 and 2017), stratified by age
- HIV prevalence data from national household surveys (in 2005, 2008, 2012, 2016 and 2017), stratified by age and sex
- Recorded numbers of deaths in adults (1997-2016), stratified by age and sex
- HIV prevalence data from studies conducted among MSM and FSWs
- National household survey data on the proportion of adults who are receiving ART (2012 and 2017), stratified by sex

For each data source, a likelihood function is specified, representing the model goodness of fit to the data. The model is calibrated using a Bayesian algorithm, with the posterior distribution being estimated by Incremental Mixture Importance Sampling.[17]

**Modelling of oral PrEP (TDF/FTC)**

*Effectiveness of oral PrEP*

Randomized controlled trials published to date have yielded conflicting estimates of the effectiveness of PrEP, mostly because of differences in PrEP adherence across trials. Although a meta-analysis estimated that PrEP reduced heterosexual transmission and transmission between MSM by 46% and 66% respectively,[18] these estimates are probably under-estimates, as most of the evidence included in the meta-analysis came from randomized trials that were conducted prior to the effectiveness of PrEP being established. More recent studies, conducted in the context of known PrEP efficacy, have generally found much higher levels of adherence and effectiveness,[19–21] suggesting that individuals are more motivated to use PrEP consistently when they know that it works. The assumed effectiveness of PrEP is therefore set to 65% in heterosexuals and 85% in MSM. The assumed effectiveness of 65% in heterosexuals is based on a meta-analysis that found an average 65% reduction in women's HIV risk in studies in which average PrEP adherence was at least 50%,[22] and the assumed effectiveness of 85% in MSM is based on the results of the PROUD and IPERGAY studies, which both found 86% effectiveness in MSM.[19,20] The assumed greater effectiveness of PrEP in MSM is supported by in vitro evidence of greater drug concentration in rectal tissue when compared to female genital tract tissue.[23] There is unfortunately relatively little data on the effectiveness of PrEP in heterosexual men, so we assume effectiveness to be the same as in MSM.

*Risk compensation*

Although data from randomized trials generally do not show evidence of risk compensation in PrEP recipients,[24–26] it is difficult to extrapolate from the data collected in these randomized trials, as trial participants would have been counselled on the uncertainty regarding the efficacy of the products that were being evaluated, and even if they believed the study products to be effective, would not have known whether they were receiving the study drug or the placebo. In an analysis of changes in behaviour after the unblinding of the Partners PrEP trial in heterosexual couples, a statistically significant 10% increase was noted in unprotected extramarital sex, amongst individuals who were receiving open-label PrEP[27] A recent meta-analysis of PrEP studies conducted in MSM also found that PrEP use was associated with increased STI diagnosis (OR 1·26, 95% CI: 0·99-1·54) and increases in condomless sex.[28] Based on these two studies, we assume a 10% reduction in condom use among PrEP users. However, it is worth noting that this assumption is subject to much uncertainty; in a more recent study of PrEP uptake in heterosexual sero-discordant couples, no reduction in condom use was observed after PrEP initiation.[29]

*Oral PrEP discontinuation*

Rates at which individuals discontinue PrEP are highly variable between studies, ranging from rates of 0·23 per annum in American MSM[30] to rates of 0·45 and 0·80 per annum in studies that have followed individuals following the completion of randomized controlled trials of PrEP.[27,31] In our model we assume an average PrEP duration based on the limited programme data available in South Africa for female sex workers (FSWs) and MSM

(Sarah Jenkins, personal communication). We fit simple Weibull models to the data to estimate the time from initiating PrEP to stopping PrEP; in the case of FSWs, a Weibull distribution with a mean of 4·8 months and a shape parameter of 0·45 provides an adequate fit to the data, while in the case of MSM, a Weibull distribution with a mean of 11·1 months and a shape parameter of 0·60 provides an adequate fit to the data (Figure S1). The model does allow previous PrEP users to re-enrol into the PrEP programme, however previous history of PrEP is not tracked separately in the model and we assume that former PrEP users initiate PrEP at the same rate as other eligible individuals.

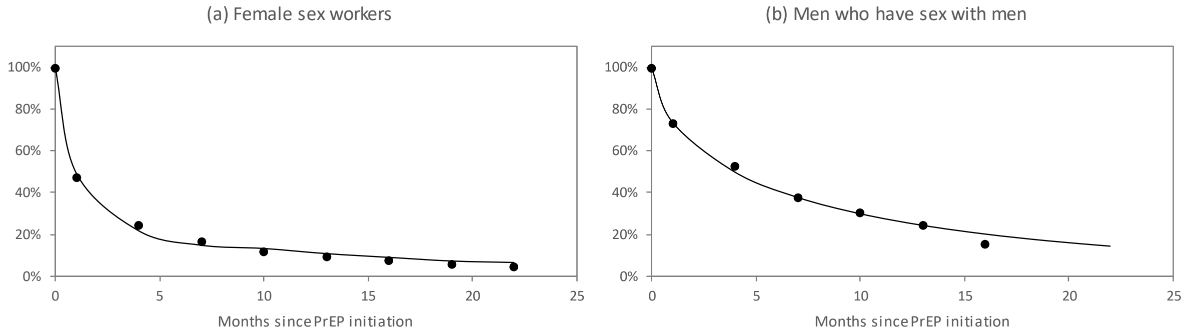


**Figure S1: Retention in South African PrEP programmes**

Data (represented by dots) are from South African PrEP programmes, as at November 2018 (Sarah Jenkins, personal communication). The solid lines represent Weibull fits to the data.

*Effect of risk group on PrEP initiation*

Initially, oral PrEP in South Africa was promoted mainly to FSWs and MSM. In recent years there has been increasing promotion of oral PrEP to adolescent girls and young women (AGYW). It is likely that high-risk AGYW initiate PrEP at a greater rate than low-risk AGYW, given that they are more likely to perceive themselves as being at high risk. However, there is a lack of local data on the predictors of PrEP uptake among AGYW. We therefore rely on a study of correlates of PrEP uptake among pregnant Kenyan women,[32] assuming those in the low-risk group are 0·33 times as likely to initiate PrEP as those in the high-risk group.

## References


1 Johnson L, Dorrington R. Thembisa version 4.4: a model for evaluating the impact of HIV/AIDS in South Africa. 2021 https://www.thembisa.org.

2 Ramjee G, Karim SS, Sturm AW. Sexually transmitted infections among sex workers in KwaZulu-Natal, South Africa. *Sex Transm Dis* 1998; **25**: 346–9.

3 Dunkle KL, Beksinska ME, Rees VH, Ballard RC, Htun Y, Wilson ML. Risk factors for HIV infection among sex workers in Johannesburg, South Africa. *Int J STD AIDS* 2005; **16**: 256–61.

4 Peltzer K, Seoka P, Raphala S. Characteristics of female sex workers and their HIV/AIDS/STI knowledge, attitudes and behaviour in semi-urban areas in South Africa. *Curationis* 2004; **27**: 4–11.

5 Simbayi LC, Marang Men's Project (South Africa), Human Sciences Research Council, editors. The South African Marang Men's Project: HIV bio-behavioural surveys conducted among men who have sex with men in Cape Town, Durban and Johannesburg, using respondent- driven sampling. Cape Town, South Africa: HSRC Press, 2014.

6 Lane T, Osmand T, Marr A, *et al.* The Mpumalanga Men's Study (MPMS): results of a baseline biological and behavioral HIV surveillance survey in two MSM communities in South Africa. *PLoS One* 2014; **9**: e111063.

7 Lane T, Raymond HF, Dladla S, *et al.* High HIV prevalence among men who have sex with men in Soweto, South Africa: results from the Soweto Men's Study. *AIDS Behav* 2011; **15**: 626–34.

8 Vu L, Tun W, Sheehy M, Nel D. Levels and correlates of internalized homophobia among men who have sex with men in Pretoria, South Africa. *AIDS Behav* 2012; **16**: 717–23.

9 Fearon E, Tenza S, Mokoena C, *et al.* HIV testing, care and viral suppression among men who have sex with men and transgender individuals in Johannesburg, South Africa. *PLoS One* 2020; **15**: e0234384.

10 Johnson L, Dorrington R, Bradshaw D, Pillay-Van Wyk V, Rehle T. Sexual behaviour patterns in South Africa and their association with the spread of HIV: insights from a mathematical model. *DemRes* 2009; **21**: 289–340.

11 Johnson LF, Hallett TB, Rehle TM, Dorrington RE. The effect of changes in condom usage and antiretroviral treatment coverage on human immunodeficiency virus incidence in South Africa: a model-based analysis. *J R Soc Interface* 2012; **9**: 1544–54.

12 Weller S, Davis K. Condom effectiveness in reducing heterosexual HIV transmission. *Cochrane Database Syst Rev* 2002; : CD003255.

13 Hughes JP, Baeten JM, Lingappa JR, *et al.* Determinants of per-coital-act HIV-1 infectivity among African HIV-1-serodiscordant couples. *J Infect Dis* 2012; **205**: 358–65.

14 Weiss HA, Halperin D, Bailey RC, Hayes RJ, Schmid G, Hankins CA. Male circumcision for HIV prevention: from evidence to action? *AIDS* 2008; **22**: 567–74.

15 Johnson LF, Rehle TM, Jooste S, Bekker L-G. Rates of HIV testing and diagnosis in South Africa: successes and challenges. *AIDS* 2015; **29**: 1401–9.

16 Jooste S, Mabaso M, Taylor M, North A, Tadokera R, Simbayi L. Trends and determinants of ever having tested for HIV among youth and adults in South Africa from 2005–2017: Results from four repeated cross-sectional nationally representative household-based HIV prevalence, incidence, and behaviour surveys. *PLoS ONE* 2020; **15**: e0232883.

17 Raftery AE, Bao L. Estimating and Projecting Trends in HIV/AIDS Generalized Epidemics Using Incremental Mixture Importance Sampling. *Biometrics* 2010; **66**: 1162–73.

18 Fonner VA, Dalglish SL, Kennedy CE, *et al.* Effectiveness and safety of oral HIV preexposure prophylaxis for all populations. *AIDS* 2016; **30**: 1973–83.

19 Molina J-M, Capitant C, Spire B, *et al.* On-Demand Preexposure Prophylaxis in Men at High Risk for HIV-1 Infection. *N Engl J Med* 2015; **373**: 2237–46.

20 McCormack S, Dunn DT, Desai M, *et al.* Pre-exposure prophylaxis to prevent the acquisition of HIV-1 infection (PROUD): effectiveness results from the pilot phase of a pragmatic open-label randomised trial. *The Lancet* 2016; **387**: 53–60.

21 Bekker L-G, Roux S, Sebastien E, *et al.* Daily and non-daily pre-exposure prophylaxis in African women (HPTN 067/ADAPT Cape Town Trial): a randomised, open-label, phase 2 trial. *Lancet HIV* 2018; **5**: e68–78.

22 Hanscom B, Janes HE, Guarino PD, *et al.* Brief Report: Preventing HIV-1 Infection in Women Using Oral Preexposure Prophylaxis: A Meta-analysis of Current Evidence. *J Acquir Immune Defic Syndr* 2016; **73**: 606–8.

23 Cottrell ML, Yang KH, Prince HMA, *et al.* A Translational Pharmacology Approach to Predicting Outcomes of Preexposure Prophylaxis Against HIV in Men and Women Using Tenofovir Disoproxil Fumarate With or Without Emtricitabine. *J Infect Dis* 2016; **214**: 55–64.

24 Baeten JM, Donnell D, Ndase P, *et al.* Antiretroviral Prophylaxis for HIV Prevention in Heterosexual Men and Women. *N Engl J Med* 2012; **367**: 399–410.

25 Thigpen MC, Kebaabetswe PM, Paxton LA, *et al.* Antiretroviral preexposure prophylaxis for heterosexual HIV transmission in Botswana. *N Engl J Med* 2012; **367**: 423–34.

26 Van Damme L, Corneli A, Ahmed K, *et al.* Preexposure Prophylaxis for HIV Infection among African Women. *N Engl J Med* 2012; **367**: 411–22.

27 Mugwanya KK, Donnell D, Celum C, *et al.* Sexual behaviour of heterosexual men and women receiving antiretroviral pre-exposure prophylaxis for HIV prevention: a longitudinal analysis. *The Lancet Infectious Diseases* 2013; **13**: 1021–8.

28 Traeger MW, Schroeder SE, Wright EJ, *et al.* Effects of Pre-exposure Prophylaxis for the Prevention of Human Immunodeficiency Virus Infection on Sexual Risk Behavior in Men Who Have Sex With Men: A Systematic Review and Meta-analysis. *Clin Infect Dis* 2018; **67**: 676–86.

29 Ortblad KF, Stalter RM, Bukusi EA, *et al.* No Evidence of Sexual Risk Compensation Following PrEP Initiation Among Heterosexual HIV Serodiscordant Couples in Kenya and Uganda. *AIDS Behav* 2020; **24**: 1365–75.

30 Liu A, Cohen S, Follansbee S, *et al.* Early experiences implementing pre-exposure prophylaxis (PrEP) for HIV prevention in San Francisco. *PLoS Med* 2014; **11**: e1001613.

31 Grant RM, Anderson PL, McMahan V, *et al.* Uptake of pre-exposure prophylaxis, sexual practices, and HIV incidence in men and transgender women who have sex with men: a cohort study. *Lancet Infect Dis* 2014; **14**: 820–9.

32 Kinuthia J, Pintye J, Mugwanya K, Serede M, Sila J, Abuna F. High PrEP uptake among Kenyan pregnant women offered PrEP during antenatal care. In: Conference on Retroviruses and Opportunistic Infections. Boston, Massachusetts, 2018.

**Table S1: Details of cost items, unit cost, quantities and their sources by visit type for CAB-LA provision, for young women** (under the assumption that the cost of one CAB-LA injection is the same price the equivalent protection period of oral TDF/FTC, i.e. 2 months' supply = $9·40, and oral CAB cost for 20% of the population is the same as oral TDF/FTC = $4·70; and average duration on CAB-LA was 5 months)

| | Cost Category | Ingredient | Unit cost (2021 USD) | Cost unit | Source* | Quantity | Quantity source/assumption | Subtotal Cost (2021 USD) |
|---|---|---|---|---|---|---|---|---|
| **Patient-level cost†** | | | | | | | | |
| **Screening/Initiation** | | | | | | | | **23·74** |
| ***Education/readiness assessment*** | | | | | | | | |
| Readiness assessment | Staff | Counsellor | 0·06 | per minute | 1 | 1 | Assumption | 0·06 |
| STI screening form | Consumables | Patient form | 0·01 | per form | 2 | 2 | Assumption | 0·02 |
| ***HIV testing (screening/initiation)*** | | | | | | | | |
| 1st test (including mark-up for people testing HIV+ that don't initiate PrEP) | Labs | HIV rapid test | 0·52 | per test | 3 | 1·128 | 1/(1-HIV prevalence) | 0·59 |
| | Staff | Counsellor | 0·06 | per minute | 1 | 16·128 | Data from demonstration projects (personal communication, Kevin Rebe/ Gaby Gomez) | 1·01 |
| | Consumables | Gloves | 0·07 | per pair | 4 | 1·128 | 1/(1-HIV prevalence) | 0·08 |
| | Consumables | Cotton wool swabs | 0·02 | per swab | 5 | 1·128 | 1/(1-HIV prevalence) | 0·03 |
| 2nd test (only if 1st positive) | Labs | HIV rapid test | 0·52 | per test | 3 | 0·114 | HIV prevalence | 0·06 |
| | Staff | Counsellor | 0·06 | per minute | 1 | 1·833 | Data from demonstration projects (personal communication, Kevin Rebe/ Gaby Gomez) | 0·11 |
| | Consumables | Gloves | 0·07 | per pair | 4 | 0·114 | HIV prevalence | 0·01 |
| | Consumables | Cotton wool swabs | 0·02 | per swab | 5 | 0·114 | HIV prevalence | 0·003 |
| Only in case of discrepant rapid tests | Labs | ELISA | 4·12 | per test | 6 | 0·02 | Assumption | 0·08 |
| | Staff | Counsellor | 0·06 | per minute | 1 | 0·3 | Assumption (15 minutes x probability of 0·02) | 0·02 |
| | Consumables | Gloves | 0·07 | per pair | 4 | 0·02 | Assumption (1 set x probability of 0·02) | 0·001 |
| | Consumables | Needle | 0·01 | per needle | 7 | 0·02 | Assumption (1 set x probability of 0·02) | 0·0002 |
| | Consumables | Cotton wool swabs | 0·02 | per swab | 5 | 0·02 | Assumption (1 set x probability of 0·02) | 0·0005 |
| ***Other monitoring tests*** | | | | | | | | |
| Blood draw and symptom check | Staff | Professional nurse | 0·29 | per minute | 1 | 15 | Assumption | 4·38 |
| Alanine aminotransferase (ALT) test | Labs | ALT test | 3·39 | per test | 6 | 1 | Baxter (2013) | 3·39 |
| Pregnancy test | Labs | Pregnancy test (urine) | 0·28 | per test | 8 | 1 | One per person | 0·28 |
| ***STI screening (syndromic management)*** | | | | | | | | |
| STI symptom screen | Staff | Counsellor | 0·06 | per minute | 1 | 5 | Assumption | 0·31 |

| | Cost Category | Ingredient | Unit cost (2021 USD) | Cost unit | Source* | Quantity | Quantity source/assumption | Subtotal Cost (2021 USD) |
|---|---|---|---|---|---|---|---|---|
| **Initial Syphilis testing** | | | | | | | | |
| Syphilis RPR | Labs | RPR titre | 2·41 | per test | 9 | 1 | One per person (or depending on syphilis prevalence for FSW, MSM) | 2·41 |
| **Counselling and assessment** | | | | | | | | |
| Adherence counselling | Staff | Counsellor | 0·06 | per minute | 1 | 15 | Data from demonstration projects (personal communication, Kevin Rebe) | 0·94 |
| **PrEP dispensing** | | | | | | | | |
| CAB-LA | Drugs | Cabotegravir injectable | 9·40 | per injection | 10 | 0·8 | One per person | 7·52 |
| Oral CAB lead in | Drugs | Oral Cabotegravir | 4·70 | per month | 10 | 0·2 | One per person | 0·94 |
| Prescribing & dispensing | Staff | Professional nurse | 0·29 | per minute | 1 | 5 | Assumption | 1·46 |
| | Consumables | Needle | 0·01 | per needle | 7 | 0·8 | Assumption | 0·01 |
| | Consumables | Cotton wool swabs | 0·02 | per swab | 5 | 0·8 | Assumption | 0·02 |
| **Annually** | | | | | | | | **2·41** |
| Syphilis RPR | Labs | RPR titre | 2·41 | per test | 9 | 1 | One per person | 2·41 |
| **Month 1 and Follow-up (all users); Month 2 (for 20% of those opting for a CAB-LA oral lead in; quantities set at 20%)** | | | | | | | | **13·80** |
| ***HIV testing (follow-up)*** | | | | | | | | |
| 1st test | Labs | HIV rapid test | 0·52 | per test | 3 | 1 | One per person | 0·52 |
| | Staff | Counsellor | 0·06 | per minute | 1 | 15 | Data from demonstration projects (personal communication, Kevin Rebe/ Gaby Gomez) | 0·94 |
| | Consumables | Gloves | 0·07 | per pair | 4 | 1 | One per person | 0·07 |
| | Consumables | Cotton wool swabs | 0·02 | per swab | 5 | 1 | One per person | 0·02 |
| 2nd test (only if 1st positive) | Labs | HIV rapid test | 0·52 | per test | 3 | 0·018 | HIV incidence | 0·01 |
| | Staff | Counsellor | 0·06 | per minute | 1 | 0·294 | Data from demonstration projects (personal communication, Kevin Rebe/ Gaby Gomez) | 0·02 |
| | Consumables | Gloves | 0·07 | per pair | 4 | 0·018 | HIV incidence | 0·001 |
| | Consumables | Cotton wool swabs | 0·02 | per swab | 5 | 0·018 | HIV incidence | 0·0004 |
| Only in case of discrepant rapid tests | Labs | ELISA | 4·12 | per test | 6 | 0·02 | Assumption | 0·08 |
| | Staff | Counsellor | 0·06 | per minute | 1 | 0·3 | Assumption (15 minutes x probability of 0·02) | 0·02 |
| | Consumables | Gloves | 0·07 | per pair | 4 | 0·02 | Assumption (1 set x probability of 0·02) | 0·001 |
| | Consumables | Needle | 0·01 | per needle | 7 | 0·02 | Assumption (1 set x probability of 0·02) | 0·0002 |
| | Consumables | Cotton wool swabs | 0·02 | per swab | 5 | 0·02 | Assumption (1 set x probability of 0·02) | 0·0005 |

| | Cost Category | Ingredient | Unit cost (2021 USD) | Cost unit | Source* | Quantity | Quantity source/assumption | Subtotal Cost (2021 USD) |
|---|---|---|---|---|---|---|---|---|
| ***Other monitoring tests*** | | | | | | | | |
| Pregnancy test | Labs | Pregnancy test (urine) | 0·28 | per test | 8 | 1 | One per woman | 0·28 |
| ***STI screening (syndromic management)*** | | | | | | | | |
| STI symptom screen | Staff | Counsellor | 0·06 | per minute | 1 | 5 | Assumption | 0·31 |
| **Counselling and assessment** | | | | | | | | |
| Adherence counselling | Staff | Counsellor | 0·06 | per minute | 1 | 10 | Data from demonstration projects (personal communication, Kevin Rebe) | 0·63 |
| **PrEP dispensing** | | | | | | | | |
| CAB-LA | Drugs | Cabotegravir injectable | 9·40 | per injection | 10 | 1 | One per person | 9·40 |
| Prescribing & dispensing | Staff | Professional nurse | 0·29 | per minute | 1 | 5 | Assumption | 1·46 |
| | Consumables | Needle | 0·01 | per needle | 7 | 1 | Assumption | 0·01 |
| | Consumables | Cotton wool swabs | 0·02 | per swab | 5 | 1 | Assumption | 0·02 |
| **Re-initiation (First and second re-initiation visits, 4 weeks later)** | | | | | | | | **3·22** |
| ***HIV testing (follow-up, see details in Month 1 visit)*** | | | | | | | | 1.69 |
| ***Other monitoring tests*** | | | | | | | | |
| Pregnancy test | Labs | Pregnancy test (urine) | 0·28 | per test | 8 | 1 | One per woman | 0·28 |
| ***STI screening (syndromic management)*** | | | | | | | | |
| STI symptom screen | Staff | Counsellor | 0·06 | per minute | 1 | 5 | Assumption | 0·31 |
| **Counselling and assessment** | | | | | | | | |
| Adherence counselling | Staff | Counsellor | 0·06 | per minute | 1 | 15 | Data from demonstration projects (personal communication, Kevin Rebe) | 0·94 |
| **Health system costs (per user per year, annually)** | | | | | | | | **10·30** |
| Training (nurses) | Staff | Professional nurse | 0·29 | per minute | 11 | 0·469 | Assumption (75min per year online training, 160 clients per yr, 1 per year) | 0·14 |
| Training (counsellors) | Staff | Counsellor | 0·06 | per minute | 11 | 0·469 | Assumption (75min per year online training, 160 clients per yr, 1 per year) | 0·03 |
| Training (peer educators) | Staff | Peer educator | 202·95 | per month | 12 | 0·000054 | Assumption (75min per year online training, 160 clients per yr, 1 per year) | 0·01 |
| Training (data capturer) | Staff | Data capturer | 0·08 | per minute | 2 | 0·469 | Assumption (75min per year online training, 160 clients per yr, 1 per year) | 0·04 |
| Training system maintenance | Overheads | Training system maintenance | 1473·11 | per year | 14 | 0·0000007 | Assumption (9,400 HCW trained,160 clients per year) | 0·0001 |
| **Mobilisation** | | | | | | | | |

| | **Cost Category** | **Ingredient** | **Unit cost (2021 USD)** | **Cost unit** | **Source*** | **Quantity** | **Quantity source/assumption** | **Subtotal Cost (2021 USD)** |
|---|---|---|---|---|---|---|---|---|
| Outreach campaign | Overheads | Outreach campaign event | 13·75 | per person | 15 | 0·010 | Assumption (proportion initiated out of all reached = 1/100) | 0·14 |
| IEC materials | Consumables | IEC material | 0·19 | per person | 13 | 1·000 | Assumption (1 set per client per year) | 0·19 |
| Outreach | Staff | Peer educator | 202·95 | per month | 12 | 0·042 | Assumption (15 mins out of 8 hrs for PrEP/160 clients per year) | 8·60 |
| **M+E** | | | | | | | | |
| Patient form | Consumables | Patient form | 0·01 | per form | 2 | 2·000 | 1 x 2-page clinical form per year per person on PrEP | 0·02 |
| Paper register (A3 page) | Consumables | Paper A3 size | 0·02 | per page | 16 | 0·075 | 1 x A3 per month per clinic | 0·00 |
| Staff time for monthly reporting | Staff | Data capturer | 0·08 | per minute | 1 | 4·500 | Assumption (60min per month/site) | 0·36 |
| **Overhead** | | | | | | | | |
| Building maintenance and utilities | Overheads | | ***7% mark-up on per visit costs, applied to overall cost*** | | | | | |
| Drawing patient file | Staff | Clerk | 0·07 | per minute | 1 | 11·4 | Assumption (3 minutes x number of visits per year) | 0·77 |

† Average cost per person initiated is structured based on the types of visits occurring given the average duration each population is on CAB-LA. All populations and duration scenarios have 1 visit for screening/initiation, 1 visit at Month 1 (first year only), 20% of patients with oral CAB-lead in have a visit at Month 2 (first year only). Re-initiation (1) and (2) only occur if clients are on CAB-LA for 12 months or longer. Follow-up visits occur 2-monthly after Month 1 visits and are dependent on the assumed average duration on CAB-LA.
‡ Health systems costs are assumed for each client per year, and training occurs once a year. In addition to these costs, a 7% overhead costs are included for each visit type under patient-level costs to cover utilities and space requirements, as per personal communication with Gaby Gomez.

**Source of unit costs:**
1. South African Government Salary Scale (2019); 2. South Africa, Global Fund proposal (2013); 3. South African National Department of Health, contract RT41-2017; 4. National Treasury of South Africa, tender RT76-2016; 5. South African National Department of Health, tender HM022015BD; 6. South African National Health Laboratory Service price list (2018); 7. South African National Department of Health, contract HM08-2015SYR; 8. Kendon Medical Supplies Pty Ltd, quote (19 Aug 2019); 9. South African National Health Laboratory Service price list (2017); 10. South African National Department of Health, contract RT71-2019; 11. Clinton Health Access Initiative (2015); 12. Provincial DOH CG Business Plans (2017); 13. Personal communication, Steve Cohen (2015); 14. Personal communication, Hasina Sebudar (2020); 15. RSA Global Fund Grant portfolio budgets (2018)

**Table S2: Probability distributions used for parameters varied in the probabilistic sensitivity analysis**

| Variable | Population | Distribution | Mean, standard deviation |
|---|---|---|---|
| Reduction in condom use while on PrEP | All populations | Beta (0·80, 7·20) | 0·10, 0·10 |
| Relative rate of uptake of PrEP in low risk group vs high risk group | Adolescent girls and young women (AGYW), adolescents boys and young men (ABYM) | Beta (1·49, 3·03) | 0·33, 0·20 |
| TDF/FTC annual initiation rates | AGYW, ABYM | Uniform(0,0·650) | 0·325,0·188 |
| | Female sex workers (FSW) | Uniform(0,2·733) | 1·367,0·789 |
| | Men who have sex with men (MSM) | Uniform(0,1·149) | 0·574,0·332 |
| CAB-LA annual initiation rates | AGYW, ABYM | Uniform(0,0·40) | 0·20, 0·013 |
| | FSW | Uniform(0,0·70) | 0·35,0·04 |
| | MSM | Uniform(0,0·70) | 0·35,0·04 |
| TDF/FTC effectiveness | AGYW and FSW | Beta (14·14,7·61) | 0·65, 0·10 |
| | MSM and ABYM | Beta (9·99, 1·76) | 0·85, 0·10 |
| CAB-LA effectiveness | All populations | Beta (3·56, 0·19) | 0·95, 0·05 |
| Cost of PrEP provision, excluding drugs (TDF/FTC) | AGYW (first year) | Gamma(12,0·23150) | 51, 15 |
| | FSW (first year) | Gamma(12,0·22450) | 53, 15 |
| | ABYM (first year) | Gamma(12,0·23469) | 51, 15 |
| | MSM (first year) | Gamma(12,0·18962) | 63, 18 |
| Cost of PrEP provision, excluding drugs (CAB-LA minimum duration scenario) | AGYW (first year) | Gamma(12,0·26502) | 45, 13 |
| | FSW (first year) | Gamma(12,0·25367) | 47, 13 |
| | ABYM (first year) | Gamma(12,0·27104) | 44, 13 |
| | MSM (first year) | Gamma(12,0·20820) | 57, 16 |
| Cost of PrEP provision, excluding drugs (CAB-LA maximum duration scenario) | AGYW (first year) | Gamma(12,0·16907) | 70, 20 |
| | FSW (first year) | Gamma(12,0·16750) | 71, 20 |
| | ABYM (first year) | Gamma(12,0·17564) | 68, 19 |
| | MSM (first year) | Gamma(12,0·18395) | 65, 19 |
| | MSM (follow-up year) | Gamma(12,0·24947) | 48, 14 |

Monte Carlo simulation methods were used to conducted with 1,000 model runs, while sampling for the above variables, from the corresponding distribution and shape parameters, for each model run. The number of simulations (N=1,000) was deemed appropriate to achieve an acceptable level of precision (standard error = 0.03), considering the aim of the analysis was to assess the sensitivity of the sampled variables on the main results.

## Table S3: Proportion of total cost of HIV programme spent to different areas

| | | | | CAB-LA | | | | | | | |
|---|---|---|---|---|---|---|---|---|---|---|---|
| | | | | **High coverage** | | | | **Medium coverage** | | | |
| | | | | **Maximum duration** | | **Minimum duration** | | **Maximum duration** | | **Minimum duration** | |
| | | **TDF/FTC** | | *CAB-LA drug cost relative to TDF/FTC* | | | | | | | |
| | **Baseline** | **High coverage** | **Medium coverage** | **x1** | **x2** | **x1** | **x2** | **x1** | **x2** | **x1** | **x2** |
| HIV care and treatment | 90% | 86% | 88% | 76% | 71% | 79% | 75% | 82% | 78% | 84% | 82% |
| Prevention | 5% | 10% | 8% | 20% | 26% | 17% | 21% | 14% | 17% | 11% | 14% |
| HIV testing | 4% | 4% | 4% | 3% | 3% | 4% | 4% | 4% | 4% | 4% | 4% |
| Prevention of mother-to-child transmission | <1% | <1% | <1% | <1% | <1% | <1% | <1% | <1% | <1% | <1% | <1% |
| Other | <1% | <1% | <1% | <1% | <1% | <1% | <1% | <1% | <1% | <1% | <1% |

***HIV care and treatment includes**: antiretroviral treatment to adults and children, inpatient hospital care to HIV positive patients, palliative care; **Prevention includes:** medical male circumcision, condom distribution, pre-exposure prophylaxis, post-exposure prophylaxis, combination prevention packages to female sex workers; **HIV testing includes**: testing of infants at birth and 10 weeks, general HIV testing at primary health care clinics, HIV testing in antenatal care clinics, provider-initiated HIV testing and counselling, mobile HIV testing, home-based HIV testing, partner notification HIV testing and HIV self-testing; **prevention of mother-to-child transmission** is restricted to mothers who are not already initiated on antiretroviral treatment. **Other includes:** supply chain management and pharmacovigilance programme.

## Table S4: Estimated cost threshold per CAB-LA injection to ensure CAB-LA remains as cost-effective as oral TDF/FTC (2021 USD)

| | **Minimum duration scenario** | | **Maximum duration scenario** | |
|---|---|---|---|---|
| **Cost per CAB-LA injection solving for** | ***Medium coverage*** | ***High coverage*** | ***Medium coverage*** | ***High coverage*** |
| *CAB-LA cost/HIV infection averted = TDF/FTC cost/HIV infection averted* | 14·47 | 11·57 | 11·79 | 9·03 |
| *CAB-LA cost/life year saved = TDF/FTC cost/life year saved* | 14·47 | 11·88 | 11·70 | 9·33 |

**Table S5: Impact and cost-effectiveness of CAB-LA over baseline* and oral PrEP over baseline, over a 20-year time horizon (2022-2041); assuming that CAB-LA injections are given 3-monthly**

| **Scenario** | **New HIV infections** | | **Life years lost due to AIDS** | | **CAB-LA drug cost relative to oral PrEP drug†** | **Total cost of the HIV programme (2021 USD)** | | **Incremental cost effectiveness (2021 USD)** | |
|---|---|---|---|---|---|---|---|---|---|
| | *Number [millions]* | *% averted over BL* | *Number [millions]* | *% saved over BL* | | *Cost [billions]* | *Incremental cost over BL* | *Cost/ infection averted* | *Cost/ life year saved* |
| **Baseline (BL)** | 3·02 | | 37·34 | | | 41·29 | | | |
| **Medium PrEP coverage** | | | | | | | | | |
| TDF/FTC | 2·89 | 4% | 37·00 | 1% | N/A | 42·08 | 2% | 6,053 | 2,309 |
| CAB-LA minimum duration | 2·58 | 15% | 36·19 | 3% | 1x | 43·35 | 5% | 4,692 | 1,790 |
| | | | | | 2x | 44·77 | 8% | 7,921 | 3,022 |
| | | | | | 3x | 46·19 | 12% | 11,150 | 4,253 |
| | | | | | 4x | 47·60 | 15% | 14,380 | 5,485 |
| | | | | | 5x | 49·02 | 19% | 17,609 | 6,717 |
| CAB-LA maximum duration | 2·44 | 19% | 35·81 | 4% | 1x | 44·18 | 7% | 4,927 | 1,889 |
| | | | | | 2x | 46·24 | 12% | 8,445 | 3,239 |
| | | | | | 3x | 48·30 | 17% | 11,963 | 4,588 |
| | | | | | 4x | 50·36 | 22% | 15,481 | 5,937 |
| | | | | | 5x | 52·42 | 27% | 18,999 | 7,286 |
| **High PrEP coverage** | | | | | | | | | |
| TDF/FTC | 2·78 | 8% | 36·68 | 2% | N/A | 42·92 | 4% | 6,610 | 2,498 |
| CAB-LA minimum duration | 2·31 | 24% | 35·41 | 5% | 1x | 45·62 | 10% | 6,052 | 2,246 |
| | | | | | 2x | 48·45 | 17% | 10,020 | 3,718 |
| | | | | | 3x | 51·29 | 24% | 13,989 | 5,191 |
| | | | | | 4x | 54·13 | 31% | 17,957 | 6,664 |
| | | | | | 5x | 56·96 | 38% | 21,926 | 8,137 |
| CAB-LA maximum duration | 2·17 | 28% | 35·03 | 6% | 1x | 46·85 | 13% | 6,496 | 2,403 |
| | | | | | 2x | 50·63 | 23% | 10,913 | 4,037 |
| | | | | | 3x | 54·41 | 32% | 15,330 | 5,671 |
| | | | | | 4x | 58·19 | 41% | 19,746 | 7,305 |
| | | | | | 5x | 61·97 | 50% | 24,163 | 8,939 |

*Baseline scenario: current roll-out of TDF/FTC as standard of care PrEP (see Table 1 for comparative coverage levels by population). † Drug cost only, excluding cost of provision (staff, lab monitoring, consumables and overhead).
Abbreviations: HIV=Human immunodeficiency virus, AIDS = acquired immunodeficiency syndrome, CAB-LA = long-acting injectable cabotegravir, USD = United States Dollars, BL = Baseline, PrEP = pre-exposure prophylaxis

**Table S6: Impact and cost-effectiveness of CAB-LA over baseline* and oral PrEP over baseline, over a 20-year time horizon (2022-2041); assuming same coverage for CAB-LA and TDF/FTC**

| Scenario | New HIV infections | | Life years lost due to AIDS | | CAB-LA drug cost relative to oral PrEP drug† | Total cost of the HIV programme (2021 USD) | | Incremental cost effectiveness (2021 USD) | |
|---|---|---|---|---|---|---|---|---|---|
| | *Number [millions]* | *% averted over BL* | *Number [millions]* | *% saved over BL* | | *Cost [billions]* | *Incremental cost over BL* | *Cost/ infection averted* | *Cost/ life year saved* |
| **Baseline (BL)** | 3·02 | | 37·34 | | | 41·29 | | | |
| **Medium PrEP coverage** | | | | | | | | | |
| TDF/FTC | 2·89 | 4% | 37·00 | 1% | N/A | 42·08 | 2% | 6,053 | 2,309 |
| CAB-LA minimum duration | 2·83 | 6% | 36·83 | 1% | 1x | 41·96 | 2% | 3,449 | 1,322 |
| | | | | | 2x | 42·41 | 3% | 5,750 | 2,203 |
| | | | | | 3x | 42·86 | 4% | 8,051 | 3,085 |
| | | | | | 4x | 43·31 | 5% | 10,352 | 3,967 |
| | | | | | 5x | 43·76 | 6% | 12,653 | 4,849 |
| CAB-LA maximum duration | 2·73 | 10% | 36·60 | 2% | 1x | 42·40 | 3% | 3,843 | 1,497 |
| | | | | | 2x | 43·16 | 5% | 6,482 | 2,524 |
| | | | | | 3x | 43·92 | 6% | 9,120 | 3,551 |
| | | | | | 4x | 44·68 | 8% | 11,758 | 4,578 |
| | | | | | 5x | 45·44 | 10% | 14,396 | 5,606 |
| **High PrEP coverage** | | | | | | | | | |
| TDF/FTC | 2·78 | 8% | 36·68 | 2% | N/A | 42·92 | 4% | 6,610 | 2,498 |
| CAB-LA minimum duration | 2·67 | 12% | 36·40 | 3% | 1x | 42·70 | 3% | 3,984 | 1,513 |
| | | | | | 2x | 43·59 | 6% | 6,490 | 2,464 |
| | | | | | 3x | 44·48 | 8% | 8,995 | 3,416 |
| | | | | | 4x | 45·37 | 10% | 11,501 | 4,367 |
| | | | | | 5x | 46·26 | 12% | 14,007 | 5,319 |
| CAB-LA maximum duration | 2·54 | 16% | 36·08 | 3% | 1x | 43·51 | 5% | 4,617 | 1,765 |
| | | | | | 2x | 44·95 | 9% | 7,621 | 2,913 |
| | | | | | 3x | 46·40 | 12% | 10,624 | 4,062 |
| | | | | | 4x | 47·84 | 16% | 13,628 | 5,210 |
| | | | | | 5x | 49·29 | 19% | 16,631 | 6,358 |

*Baseline scenario: current roll-out of TDF/FTC as standard of care PrEP (see Table 1 for comparative coverage levels by population).
† Drug cost only, excluding cost of provision (staff, lab monitoring, consumables and overhead).
Abbreviations: HIV=Human immunodeficiency virus, AIDS = acquired immunodeficiency syndrome, CAB-LA = long-acting injectable cabotegravir, USD = United States Dollars, BL = Baseline, PrEP = pre-exposure prophylaxis

**Table S7: Impact and cost-effectiveness of CAB-LA over baseline* and oral PrEP over baseline, over a 20-year time horizon (2022-2041); assuming a 3% discount rate**

| **Scenario** | **New HIV infections** | | **Life years lost due to AIDS** | | **CAB-LA drug cost relative to oral PrEP drug†** | **Total cost of the HIV programme (2021 USD)** | | **Incremental cost effectiveness (2021 USD)** | |
|---|---|---|---|---|---|---|---|---|---|
| | *Number [millions]* | *% averted over BL* | *Number [millions]* | *% saved over BL* | | *Cost [billions]* | *Incremental cost over BL* | *Cost/ infection averted* | *Cost/ life year saved* |
| **Baseline (BL)** | 2·37 | | 17·33 | | | 31·27 | | | |
| **Medium PrEP coverage** | | | | | | | | | |
| TDF/FTC | 2·28 | 4% | 17·22 | 1% | N/A | 31·86 | 2% | 6,313 | 5,264 |
| CAB-LA minimum duration | 2·23 | 6% | 17·16 | 1% | 1x | 31·78 | 2% | 3,624 | 3,039 |
| | | | | | 2x | 32·11 | 3% | 5,961 | 4,998 |
| | | | | | 3x | 32·43 | 4% | 8,297 | 6,958 |
| | | | | | 4x | 32·76 | 5% | 10,634 | 8,918 |
| | | | | | 5x | 33·09 | 6% | 12,971 | 10,877 |
| CAB-LA maximum duration | 2·17 | 9% | 17·09 | 1% | 1x | 32·11 | 3% | 4,028 | 3,431 |
| | | | | | 2x | 32·66 | 4% | 6,705 | 5,711 |
| | | | | | 3x | 33·22 | 6% | 9,382 | 7,991 |
| | | | | | 4x | 33·77 | 8% | 12,058 | 10,271 |
| | | | | | 5x | 34·32 | 10% | 14,735 | 12,551 |
| **High PrEP coverage** | | | | | | | | | |
| TDF/FTC | 2·20 | 7% | 17·12 | 1% | N/A | 32·48 | 4% | 6,847 | 5,665 |
| CAB-LA minimum duration | 2·12 | 11% | 17·02 | 1x | 1x | 32·34 | 3% | 4,149 | 3,461 |
| | | | | 2x | 2x | 32·98 | 5% | 6,680 | 5,571 |
| | | | | 3x | 3x | 33·63 | 8% | 9,210 | 7,681 |
| | | | | 4x | 4x | 34·28 | 10% | 11,740 | 9,791 |
| | | | | 5x | 5x | 34·92 | 12% | 14,270 | 11,902 |
| CAB-LA maximum duration | 2·02 | 15% | 16·92 | 2% | 1x | 32·94 | 5% | 4,789 | 4,028 |
| | | | | | 2x | 33·99 | 9% | 7,817 | 6,575 |
| | | | | | 3x | 35·04 | 12% | 10,845 | 9,122 |
| | | | | | 4x | 36·09 | 15% | 13,873 | 11,669 |
| | | | | | 5x | 37·15 | 19% | 16,901 | 14,216 |

*Baseline scenario: current roll-out of TDF/FTC as standard of care PrEP (see Table 1 for comparative coverage levels by population).
† Drug cost only, excluding cost of provision (staff, lab monitoring, consumables and overhead).
Abbreviations: HIV=Human immunodeficiency virus, AIDS = acquired immunodeficiency syndrome, CAB-LA = long-acting injectable cabotegravir, USD = United States Dollars, BL = Baseline, PrEP = pre-exposure prophylaxis

**Table S8: Impact and cost-effectiveness of CAB-LA over baseline* and oral PrEP over baseline, over a 20-year time horizon (2022-2041); assuming a 4.75% discount rate ᵖ**

| Scenario | New HIV infections | | Life years lost due to AIDS | | CAB-LA drug cost relative to oral PrEP drug† | Total cost of the HIV programme (2021 USD) | | Incremental cost effectiveness (2021 USD) | |
|---|---|---|---|---|---|---|---|---|---|
| | *Number [millions]* | *% averted over BL* | *Number [millions]* | *% saved over BL* | | *Cost [billions]* | *Incremental cost over BL* | *Cost/ infection averted* | *Cost/ life year saved* |
| **Baseline (BL)** | 2·08 | | 11·93 | | | 26·87 | | | |
| **Medium PrEP coverage** | | | | | | | | | |
| TDF/FTC | 2·00 | 4% | 11·87 | 1% | N/A | 27·36 | 2% | 6,479 | 7,764 |
| CAB-LA minimum duration | 1·96 | 6% | 11·83 | 1% | 1x | 27·30 | 2% | 3,732 | 4,497 |
| | | | | | 2x | 27·58 | 3% | 6,093 | 7,342 |
| | | | | | 3x | 27·85 | 4% | 8,454 | 10,187 |
| | | | | | 4x | 28·13 | 5% | 10,815 | 13,032 |
| | | | | | 5x | 28·40 | 6% | 13,176 | 15,876 |
| CAB-LA maximum duration | 1·91 | 8% | 11·79 | 1% | 1x | 27·58 | 3% | 4,144 | 5,070 |
| | | | | | 2x | 28·05 | 4% | 6,847 | 8,378 |
| | | | | | 3x | 28·51 | 6% | 9,550 | 11,685 |
| | | | | | 4x | 28·97 | 8% | 12,253 | 14,993 |
| | | | | | 5x | 29·44 | 10% | 14,957 | 18,300 |
| **High PrEP coverage** | | | | | | | | | |
| TDF/FTC | 1·93 | 7% | 11·81 | 1% | N/A | 27·89 | 4% | 6,997 | 8,331 |
| CAB-LA minimum duration | 1·87 | 10% | 11·75 | 1% | 1x | 27·78 | 3% | 4,252 | 5,109 |
| | | | | | 2x | 28·32 | 5% | 6,800 | 8,169 |
| | | | | | 3x | 28·86 | 7% | 9,347 | 11,230 |
| | | | | | 4x | 29·40 | 9% | 11,894 | 14,290 |
| | | | | | 5x | 29·94 | 11% | 14,441 | 17,350 |
| CAB-LA maximum duration | 1·79 | 14% | 11·69 | 2% | 1x | 28·29 | 5% | 4,897 | 5,939 |
| | | | | | 2x | 29·17 | 9% | 7,944 | 9,632 |
| | | | | | 3x | 30·05 | 12% | 10,990 | 13,326 |
| | | | | | 4x | 30·93 | 15% | 14,036 | 17,020 |
| | | | | | 5x | 31·81 | 18% | 17,082 | 20,714 |

*Baseline scenario: current roll-out of TDF/FTC as standard of care PrEP (see Table 1 for comparative coverage levels by population).
† Drug cost only, excluding cost of provision (staff, lab monitoring, consumables and overhead).
ᵖ 4·75% is the South African repurchase rate as of 14 June 2022 (South African Reserve Bank, https://www.resbank.co.za/en/home/what-we-do/statistics/key-statistics/current-market-rates)
Abbreviations: HIV=Human immunodeficiency virus, AIDS = acquired immunodeficiency syndrome, CAB-LA = long-acting injectable cabotegravir, USD = United States Dollars, BL = Baseline, PrEP = pre-exposure prophylaxis

**Table S9: Impact and cost-effectiveness of CAB-LA over baseline* and oral PrEP over baseline, over a 20-year time horizon (2022-2041); assuming a 6% discount rate**

| **Scenario** | **New HIV infections** | | **Life years lost due to AIDS** | | **CAB-LA drug cost relative to oral PrEP drug†** | **Total cost of the HIV programme (2021 USD)** | | **Incremental cost effectiveness (2021 USD)** | |
|---|---|---|---|---|---|---|---|---|---|
| | *Number [millions]* | *% averted over BL* | *Number [millions]* | *% saved over BL* | | *Cost [billions]* | *Incremental cost over BL* | *Cost/ infection averted* | *Cost/ life year saved* |
| **Baseline (BL)** | 1·90 | | 9·38 | | | 24·23 | | | |
| **Medium PrEP coverage** | | | | | | | | | |
| TDF/FTC | 1·84 | 3% | 9·34 | <1% | N/A | 24·67 | 2% | 6,602 | 9,934 |
| CAB-LA minimum duration | 1·80 | 5% | 9·31 | 1% | 1x | 24·62 | 2% | 3,812 | 5,764 |
| | | | | | 2x | 24·86 | 3% | 6,192 | 9,362 |
| | | | | | 3x | 25·10 | 4% | 8,571 | 12,960 |
| | | | | | 4x | 25·34 | 5% | 10,951 | 16,558 |
| | | | | | 5x | 25·58 | 6% | 13,330 | 20,157 |
| CAB-LA maximum duration | 1·75 | 8% | 9·28 | 1% | 1x | 24·87 | 3% | 4,230 | 6,493 |
| | | | | | 2x | 25·28 | 4% | 6,954 | 10,674 |
| | | | | | 3x | 25·69 | 6% | 9,677 | 14,855 |
| | | | | | 4x | 26·10 | 8% | 12,401 | 19,036 |
| | | | | | 5x | 26·51 | 9% | 15,125 | 23,217 |
| **High PrEP coverage** | | | | | | | | | |
| TDF/FTC | 1·78 | 7% | 9·30 | 1% | N/A | 25·14 | 4% | 7,109 | 10,637 |
| CAB-LA minimum duration | 1·72 | 10% | 9·26 | 1% | 1x | 25·04 | 3% | 4,328 | 6,538 |
| | | | | | 2x | 25·52 | 5% | 6,889 | 10,406 |
| | | | | | 3x | 26·00 | 7% | 9,450 | 14,274 |
| | | | | | 4x | 26·47 | 9% | 12,011 | 18,143 |
| | | | | | 5x | 26·95 | 11% | 14,572 | 22,011 |
| CAB-LA maximum duration | 1·65 | 13% | 9·22 | 2% | 1x | 25·50 | 5% | 4,978 | 7,593 |
| | | | | | 2x | 26·28 | 8% | 8,039 | 12,262 |
| | | | | | 3x | 27·06 | 12% | 11,101 | 16,931 |
| | | | | | 4x | 27·83 | 15% | 14,162 | 21,600 |
| | | | | | 5x | 28·61 | 18% | 17,223 | 26,270 |

*Baseline scenario: current roll-out of TDF/FTC as standard of care PrEP (see Table 1 for comparative coverage levels by population).
† Drug cost only, excluding cost of provision (staff, lab monitoring, consumables and overhead).
Abbreviations: HIV=Human immunodeficiency virus, AIDS = acquired immunodeficiency syndrome, CAB-LA = long-acting injectable cabotegravir, USD = United States Dollars, BL = Baseline, PrEP = pre-exposure prophylaxis

**Table S10: Impact and cost-effectiveness of CAB-LA over baseline* and oral PrEP over baseline, over a 20-year time horizon (2022-2041); assuming the HIV diagnostic algorithm under CAB-LA scenarios requires polymerase chain reaction (PCR) testing annually, and at every injection visit**

| **Scenario** | **New HIV infections** | | **Life years lost due to AIDS** | | **CAB-LA drug cost relative to oral PrEP drug†** | **Total cost of the HIV programme (2021 USD)** | | **Incremental cost effectiveness (2021 USD)** | |
|---|---|---|---|---|---|---|---|---|---|
| | *Number [millions]* | *% averted over BL* | *Number [millions]* | *% saved over BL* | | *Cost [billions]* | *Incremental cost over BL* | *Cost/ infection averted* | *Cost/ life year saved* |
| **Baseline (BL)** | 3·02 | | 37·34 | | | 41·29 | | | |
| **TDF/FTC** | | | | | | | | | |
| Medium PrEP coverage | 2·89 | 4% | 37·00 | 1% | N/A | 42·08 | 2% | 6,053 | 2,309 |
| High PrEP coverage | 2·78 | 8% | 36·68 | 2% | N/A | 42·92 | 4% | 6,610 | 2,498 |
| **Annual PCR testing in CAB-LA scenarios** | | | | | | | | | |
| **Medium PrEP coverage** | | | | | | | | | |
| CAB-LA minimum duration | 2·58 | 15% | 36·19 | 3% | 1x | 44·39 | 8% | 7,069 | 2,697 |
| | | | | | 2x | 45·60 | 10% | 9,809 | 3,742 |
| | | | | | 3x | 46·80 | 13% | 12,550 | 4,787 |
| | | | | | 4x | 48·00 | 16% | 15,290 | 5,833 |
| | | | | | 5x | 49·21 | 19% | 18,031 | 6,878 |
| CAB-LA maximum duration | 2·44 | 19% | 35·81 | 4% | 1x | 46·19 | 12% | 8,363 | 3,207 |
| | | | | | 2x | 48·11 | 17% | 11,653 | 4,469 |
| | | | | | 3x | 50·04 | 21% | 14,943 | 5,731 |
| | | | | | 4x | 51·97 | 26% | 18,233 | 6,992 |
| | | | | | 5x | 53·89 | 31% | 21,523 | 8,254 |
| **High PrEP coverage** | | | | | | | | | |
| CAB-LA minimum duration | 2·31 | 24% | 35·41 | 5% | 1x | 47·69 | 15% | 8,954 | 3,323 |
| | | | | | 2x | 50·10 | 21% | 12,321 | 4,572 |
| | | | | | 3x | 52·50 | 27% | 15,689 | 5,822 |
| | | | | | 4x | 54·91 | 33% | 19,057 | 7,072 |
| | | | | | 5x | 57·32 | 39% | 22,425 | 8,322 |
| CAB-LA maximum duration | 2·17 | 28% | 35·03 | 6% | 1x | 50·53 | 22% | 10,798 | 3,995 |
| | | | | | 2x | 54·06 | 31% | 14,928 | 5,523 |
| | | | | | 3x | 57·60 | 39% | 19,058 | 7,051 |
| | | | | | 4x | 61·13 | 48% | 23,188 | 8,578 |
| | | | | | 5x | 64·67 | 57% | 27,318 | 10,106 |
| **PCR testing 2-monthly in CAB-LA scenarios** | | | | | | | | | |
| **Medium PrEP coverage** | | | | | | | | | |
| CAB-LA minimum duration | 2·58 | 15% | 36·19 | 3% | 1x | 46·35 | 12% | 11,524 | 4,396 |
| | | | | | 2x | 47·55 | 15% | 14,264 | 5,441 |
| | | | | | 3x | 48·76 | 18% | 17,005 | 6,487 |
| | | | | | 4x | 49·96 | 21% | 19,745 | 7,532 |
| | | | | | 5x | 51·16 | 24% | 22,485 | 8,577 |
| CAB-LA maximum duration | 2·44 | 19% | 35·81 | 4% | 1x | 50·60 | 23% | 15,893 | 6,095 |
| | | | | | 2x | 52·52 | 27% | 19,183 | 7,357 |
| | | | | | 3x | 54·45 | 32% | 22,473 | 8,619 |
| | | | | | 4x | 56·38 | 37% | 25,763 | 9,880 |

| Scenario | New HIV infections | | Life years lost due to AIDS | | CAB-LA drug cost relative to oral PrEP drug† | Total cost of the HIV programme (2021 USD) | | Incremental cost effectiveness (2021 USD) | |
|---|---|---|---|---|---|---|---|---|---|
| | *Number [millions]* | *% averted over BL* | *Number [millions]* | *% saved over BL* | | *Cost [billions]* | *Incremental cost over BL* | *Cost/ infection averted* | *Cost/ life year saved* |
| | | | | | 5x | 58·30 | 41% | 29,053 | 11,142 |
| **High PrEP coverage** | | | | | | | | | |
| CAB-LA minimum duration | 2·31 | 24% | 35·41 | 5% | 1x | 51·60 | 25% | 14,427 | 5,354 |
| | | | | | 2x | 54·01 | 31% | 17,795 | 6,604 |
| | | | | | 3x | 56·42 | 37% | 21,163 | 7,853 |
| | | | | | 4x | 58·82 | 42% | 24,530 | 9,103 |
| | | | | | 5x | 61·23 | 48% | 27,898 | 10,353 |
| CAB-LA maximum duration | 2·17 | 28% | 35·03 | 6% | 1x | 58·61 | 42% | 20,245 | 7,490 |
| | | | | | 2x | 62·15 | 51% | 24,374 | 9,017 |
| | | | | | 3x | 65·68 | 59% | 28,504 | 10,545 |
| | | | | | 4x | 69·22 | 68% | 32,634 | 12,073 |
| | | | | | 5x | 72·75 | 76% | 36,764 | 13,601 |

*Baseline scenario: current roll-out of TDF/FTC as standard of care PrEP (see Table 1 for comparative coverage levels by population).
† Drug cost only, excluding cost of provision (staff, lab monitoring, consumables and overhead).
Abbreviations: HIV=Human immunodeficiency virus, AIDS = acquired immunodeficiency syndrome, CAB-LA = long-acting injectable cabotegravir, USD = United States Dollars, BL = Baseline, PrEP = pre-exposure prophylaxis

**Note on analysis**

The average cost of the CAB-LA scenarios was modified to include polymerase chain reaction (PCR) testing in the HIV diagnostic algorithm in two different scenarios: 1) annually, and 2) 2-monthly. PCR testing is substantially more expensive at $28/test compared to rapid HIV testing as assumed in the main analysis ($0.52/test). Under the annual scenario we assume a PCR test was conducted at screening into the CAB-LA programme, and annually thereafter. Under the 2-monthly scenario we assume a PCR test was conducted at screening, and 2-monthly thereafter.

Incorporating PCR testing, the average cost of CAB-LA provision ranges between $110/user (young men, minimum duration; 40% more expensive than the corresponding scenario/population using HIV rapid testing) to $273 (female sex workers, maximum duration; 99% more expensive) under the annual PCR scenario; it ranges between $167/user (young men, minimum duration; 113% more expensive) to $422 (female sex workers, maximum duration; 207% more expensive) under the 2-monthly PCR scenario. The cost-effectiveness analysis results are presented in Table S10. Assuming PCR testing was part of the HIV diagnostic algorithm on an annual basis, for CAB-LA to be as cost-effective as TDF/FTC, the cost of the CAB-LA injection would need to be $0.49 (maximum duration, high coverage), $3.23 (maximum duration, medium coverage), $3.29 (minimum duration, high coverage) and $6.14 (minimum duration, medium coverage). Under the scenario where PCR testing was 2-monthly, CAB-LA would be less cost-effective than TDF/FTC under all scenarios, irrespective of the cost of the CAB-LA injection.

**Table S11: Uncertainty ranges around the impact and cost-effectiveness of CAB-LA and oral PrEP over baseline, over a 20-year time horizon (2022-2041); based on 1,000 Monte Carlo simulations in a probabilistic sensitivity analysis*;** *figures represent the median estimate with interquartile range in round brackets, and 2.5$^{th}$ and 97.5$^{th}$ percentiles in square brackets.*

| **Scenario** | **New HIV infections** | | **Life years lost due to AIDS** | | **CAB-LA drug cost relative to oral PrEP drug†** | **Total cost of the HIV programme (2021 USD)** | | **Incremental cost effectiveness (2021 USD)** | |
|---|---|---|---|---|---|---|---|---|---|
| | *Number [millions]* | *% averted over BL* | *Number [millions]* | *% saved over BL* | | *Cost [billions]* | *Incremental cost over BL* | *Cost/ infection averted* | *Cost/ life year saved* |
| **Baseline (BL)** | 3·03<br>(2·91-3·13)<br>[2·67-3·34] | | 37·46<br>(36·44-38·32)<br>[34·81-39·80] | | | 41·28<br>(40·81-41·66)<br>[40·04-42·48] | | | |
| TDF/FTC | 2·79<br>(2·71-2·89)<br>[2·62-3·01] | 8%<br>(4-10%)<br>[1-13%] | 36·70<br>(36·51-36·99)<br>[36·26-37·30] | 2%<br>(1-2%)<br>[0-3%] | N/A | 42·96<br>(42·15-43·90)<br>[41·44-45·60] | 4%<br>(2-6%)<br>[0-9%] | 7,532<br>(5,884-9,732)<br>[3,585-17,352] | 2,843<br>(2,223-3,659)<br>[1,374-6,564] |
| CAB-LA minimum duration | 2·55<br>(2·43-2·73)<br>[2·29-2·98] | 16%<br>(10-20%)<br>[2-24%] | 36·08<br>(35·73-36·57)<br>[35·32-37·22] | 3%<br>(2-4%)<br>[0-5%] | 1x | 43·70<br>(42·51-45·00)<br>[41·45-48·05] | 6%<br>(3-8%)<br>[0-14%] | 5,323<br>(4,062-6,842)<br>[2,234-9,986] | 1,986<br>(1,528-2,537)<br>[844-3,711] |
| | | | | | 2x | 45·24<br>(43·38-47·23)<br>[41·60-51·30] | 9%<br>(5-13%)<br>[1-20%] | 8,661<br>(6,859-10,744)<br>[4,412-14,853] | 3,242<br>(2,584-3,999)<br>[1,678-5,474] |
| | | | | | 3x | 46·80<br>(44·20-49·40)<br>[41·74-54·74] | 12%<br>(7-16%)<br>[1-25%] | 11,923<br>(9,641-14,662)<br>[6,566-19,736] | 4,494<br>(3,632-5,441)<br>[2,517-7,264] |
| | | | | | 4x | 48·35<br>(45·08-51·51)<br>[41·87-58·28] | 15%<br>(8-20%)<br>[1-29%] | 15,279<br>(12,411-18,531)<br>[8,709-24,693] | 5,718<br>(4,681-6,869)<br>[3,325-9,053] |
| | | | | | 5x | 49·87<br>(45·92-53·64)<br>[42·01-61·73] | 17%<br>(10-23%)<br>[2-33%] | 18,603<br>(15,194-22,371)<br>[10,885-29,924] | 6,967<br>(5,750-8,330)<br>[4,150-10,953] |
| CAB-LA maximum duration | 2·41<br>(2·28-2·61)<br>[2·15-2·94] | 20%<br>(14-24%)<br>[3-29%] | 35·70<br>(35·34-36·26)<br>[34·93-37·13] | 4%<br>(3-5%)<br>[1-6%] | 1x | 44·76<br>(43·15-46·44)<br>[41·58-50·04] | 8%<br>(4-11%)<br>[1-17%] | 5,847<br>(4,404-7,458)<br>[2,425-10,804] | 2,184<br>(1,667-2,779)<br>[938-3,985] |
| | | | | | 2x | 47·09<br>(44·53-49·76)<br>[41·83-54·57] | 12%<br>(7-17%)<br>[1-24%] | 9,765<br>(7,658-11,902)<br>[4,780-16,372] | 3,673<br>(2,880-4,455)<br>[1,852-6,047] |

| Scenario | New HIV infections | | Life years lost due to AIDS | | CAB-LA drug cost relative to oral PrEP drug† | Total cost of the HIV programme (2021 USD) | | Incremental cost effectiveness (2021 USD) | |
|---|---|---|---|---|---|---|---|---|---|
| | *Number [millions]* | *% averted over BL* | *Number [millions]* | *% saved over BL* | | *Cost [billions]* | *Incremental cost over BL* | *Cost/ infection averted* | *Cost/ life year saved* |
| | | | | | 3x | 49·38<br>(45·87-52·97)<br>[42·06-59·58] | 16%<br>(10-22%)<br>[2-31%] | 13,575<br>(10,810-16,556)<br>[7,093-22,177] | 5,123<br>(4,112-6,161)<br>[2,745-8,148] |
| | | | | | 4x | 51·77<br>(47·27-56·21)<br>[42·30-64·43] | 20%<br>(13-27%)<br>[2-36%] | 17,389<br>(14,007-21,168)<br>[9,438-27,913] | 6,545<br>(5,321-7,835)<br>[3,660-10,212] |
| | | | | | 5x | 54·18<br>(48·65-59·44)<br>[42·54-69·51] | 24%<br>(15-31%)<br>[3-41%] | 21,226<br>(17,156-25,786)<br>[11,772-33,580] | 7,993<br>(6,529-9,575)<br>[4,538-12,398] |

*Key model parameters which would influence the results were sampled from pre-determined distributions for each of the 1,000 model runs (see Table S2): intervention effectiveness for both TDF/FTC and CAB-LA, reduction in condom use while on PrEP, annual initiation rate for TDF/FTC, relative annual initiation rate for CAB-LA (to ensure a value consistently higher than the corresponding TDF/FTC scenario), relative rate of PrEP initiation in low-risk heterosexuals (i.e. those with no propensity for concurrent partnerships or commercial sex), and cost of PrEP provision (excluding drug cost).

† Drug cost only, excluding cost of provision (staff, lab monitoring, consumables and overhead).

Abbreviations: HIV=Human immunodeficiency virus, AIDS = acquired immunodeficiency syndrome, CAB-LA = long-acting injectable cabotegravir, USD = United States Dollars, BL = Baseline, PrEP = pre-exposure prophylaxis

**Table S12: Partial rank correlation coefficients of results from probabilistic sensitivity analysis, by scenario**

| Parameter | Scenario | Cost per HIV infection averted (2022-2041) | HIV infections averted (2022-2041) | Cost per CAB-LA injection threshold price |
|---|---|---|---|---|
| Reduction in condom use while on PrEP | CAB-LA maximum duration | 0·02 | -0·06 | 0·10 |
| | CAB-LA minimum duration | 0·03 | -0·07 | 0·13 |
| | TDF/FTC | 0·10 | -0·18 | N/A |
| Relative rate of PrEP uptake by those at low risk of HIV | CAB-LA maximum duration | 0·94 | 0·20 | -0·01 |
| | CAB-LA minimum duration | 0·95 | 0·20 | -0·01 |
| | TDF/FTC | 0·73 | 0·24 | N/A |
| PrEP initiation rates in AGYW and ABYM | CAB-LA maximum duration | 0·66 | 0·92 | -0·50 |
| | CAB-LA minimum duration | 0·62 | 0·95 | -0·54 |
| | TDF/FTC | 0·08 | 0·98 | N/A |
| PrEP initiation rates in FSW | CAB-LA maximum duration | 0·93 | 0·93 | -0·50 |
| | CAB-LA minimum duration | 0·93 | 0·95 | -0·54 |
| | TDF/FTC | 0·10 | 0·98 | N/A |
| PrEP initiation rates in MSM | CAB-LA maximum duration | 0·93 | 0·93 | -0·50 |
| | CAB-LA minimum duration | 0·93 | 0·95 | -0·54 |
| | TDF/FTC | 0·10 | 0·98 | N/A |
| PrEP efficacy in MSM and ABYM | CAB-LA maximum duration | -0·54 | 0·28 | -0·47 |
| | CAB-LA minimum duration | -0·61 | 0·30 | -0·56 |
| | TDF/FTC | -0·67 | 0·79 | N/A |
| PrEP efficacy in AGYW and FSW | CAB-LA maximum duration | -0·54 | 0·28 | -0·47 |
| | CAB-LA minimum duration | -0·61 | 0·30 | -0·56 |
| | TDF/FTC | -0·64 | 0·79 | N/A |
| Average cost of PrEP provision for MSM | CAB-LA maximum duration | 0·91 | 0·01 | 0·32 |
| | CAB-LA minimum duration | 0·94 | 0·01 | 0·41 |
| | TDF/FTC | 0·64 | 0·03 | N/A |
| Average cost of PrEP provision for AGYW, ABYM and FSW | CAB-LA maximum duration | 0·91 | 0·01 | 0·32 |
| | CAB-LA minimum duration | 0·94 | 0·01 | 0·41 |
| | TDF/FTC | 0·64 | 0·03 | N/A |

**Figure S1. Probabilistic sensitivity analysis results comparing the incremental cost per life year saved over 2022-41 across simulations for TDF/FTC to (A) the CAB-LA minimum duration scenario and (B) the CAB-LA maximum duration scenario assuming CAB-LA is 2-fold the cost of TDF/FTC; comparing HIV infections averted over 2022-41 in TDF/FTC to CAB-LA (C) minimum duration scenario and (D) maximum duration scenario**

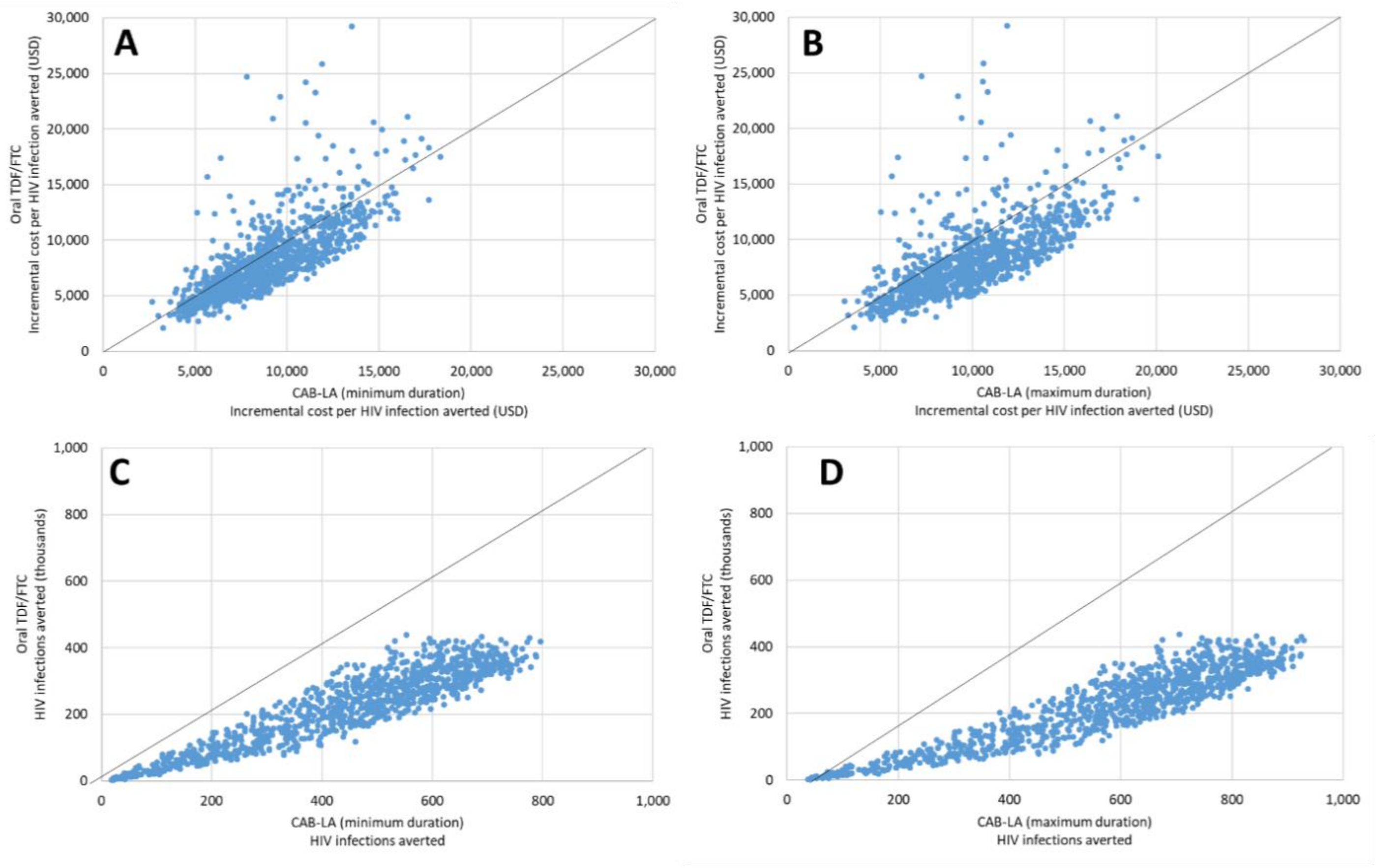


**Figure S2: Estimated cost per injection for CAB-LA as calculated based on probabilistic sensitivity analysis**

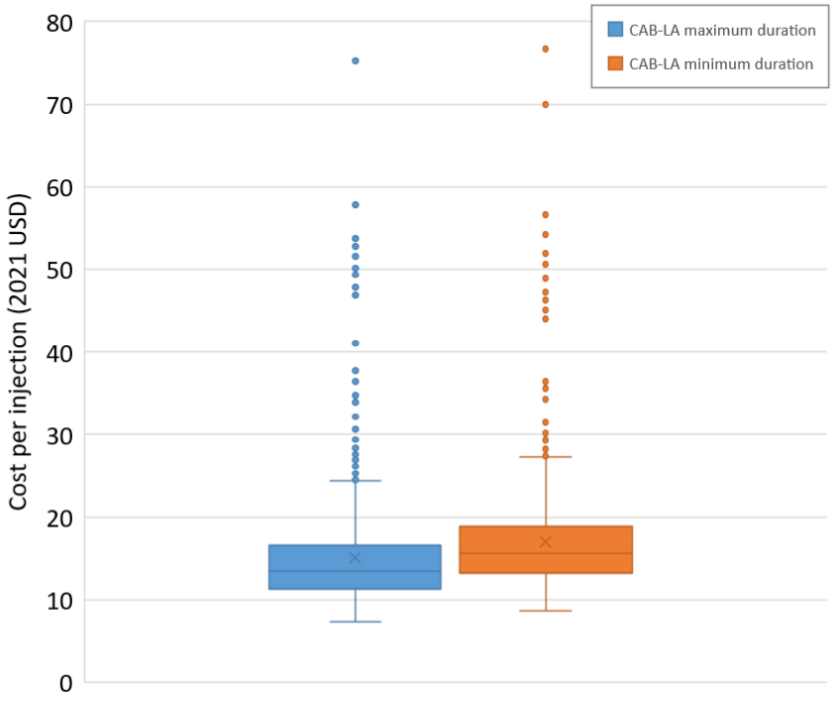

Across all simulations, the cost per CAB-LA injection was a median of \$15·60 (interquartile range (IQR) \$13·30-\$18·90, 2·5th percentile \$10·40, 97·5th percentile \$30·80) under the minimum duration scenario and \$13·50 (IQR \$11·30-\$16·60), 2·5th percentile \$8·80, 97·5th percentile \$29·70 under the maximum duration scenario·
